# Resilient microorganisms in dust samples of the International Space Station—survival of the adaptation specialists

**DOI:** 10.1186/s40168-016-0217-7

**Published:** 2016-12-20

**Authors:** Maximilian Mora, Alexandra Perras, Tatiana A. Alekhova, Lisa Wink, Robert Krause, Alina Aleksandrova, Tatiana Novozhilova, Christine Moissl-Eichinger

**Affiliations:** 1 Department for Internal Medicine, Section of Infectious Diseases and Tropical Medicine, Medical University of Graz, Auenbruggerplatz 15, 8036 Graz, Austria; 2 Department for Microbiology, University of Regensburg, Universitätsstr. 31, 93053 Regensburg, Germany; 3 Lomonosov Moscow State University, Leninskie Gory, 119991 Moscow, Russia; 4 BioTechMed Graz, Krenngasse 37, 8010 Graz, Austria

**Keywords:** International Space Station, Microbiome, Confined habitat, Archaea, Extremotolerant

## Abstract

**Background:**

The International Space Station (ISS) represents a unique biotope for the human crew but also for introduced microorganisms. Microbes experience selective pressures such as microgravity, desiccation, poor nutrient-availability due to cleaning, and an increased radiation level. We hypothesized that the microbial community inside the ISS is modified by adapting to these stresses.

For this reason, we analyzed 8–12 years old dust samples from Russian ISS modules with major focus on the long-time surviving portion of the microbial community. We consequently assessed the cultivable microbiota of these samples in order to analyze their extremotolerant potential against desiccation, heat-shock, and clinically relevant antibiotics. In addition, we studied the bacterial and archaeal communities from the stored Russian dust samples via molecular methods (next-generation sequencing, NGS) and compared our new data with previously derived information from the US American ISS dust microbiome.

**Results:**

We cultivated and identified in total 85 bacterial, non-pathogenic isolates (17 different species) and 1 fungal isolate from the 8–12 year old dust samples collected in the Russian segment of the ISS. Most of these isolates exhibited robust resistance against heat-shock and clinically relevant antibiotics. Microbial 16S rRNA gene and archaeal 16S rRNA gene targeting Next Generation Sequencing showed signatures of human-associated microorganisms (*Corynebacterium*, *Staphylococcus*, *Coprococcus* etc.), but also specifically adapted extremotolerant microorganisms. Besides bacteria, the detection of archaeal signatures in higher abundance was striking.

**Conclusions:**

Our findings reveal (i) the occurrence of living, hardy microorganisms in archived Russian ISS dust samples, (ii) a profound resistance capacity of ISS microorganisms against environmental stresses, and (iii) the presence of archaeal signatures on board. In addition, we found indications that the microbial community in the Russian segment dust samples was different to recently reported US American ISS microbiota.

**Electronic supplementary material:**

The online version of this article (doi:10.1186/s40168-016-0217-7) contains supplementary material, which is available to authorized users.

## Background

The International Space Station (ISS) is a highly unusual working place. Completely sealed off from the outside, crews of three to ten astronauts and cosmonauts have routinely inhabited the modules since 2000 -as have billions of microorganisms. The ISS represents the most confined, man-made inhabited environment to date, characterized by radiation levels higher than on Earth, low nutrient levels due to reduced introduction of organic material, constant temperature (approx. 22 °C), stable humidity (approx. 60%), and microgravity [1].

The majority of the microorganisms detected on board the ISS are human-associated (as reviewed in [1, 2]) and the ISS microbiome thus resembles the microbiome of indoor environments on Earth [3]. Next to human-derived (opportunistic) pathogens [4, 5], also technophilic microorganisms, which are able to corrode spacecraft materials [6–9], potentially inhabit the interior of the ISS. Therefore, the ISS microbial community is under constant surveillance to ensure the health of the human crew working on-board, as well as to evaluate potential risk factors for the integrity of the ISS materials and its function.

NASA (National Aeronautics and Space Administration) has identified that knowledge of the ISS microbiome is a major target for ongoing and future research studies. There is particular interest on the response of microbial communities to selective pressures such as microgravity, which could induce severe changes and adaptation processes [10].

Recent studies assessed the ISS dust microbiota using next-generation sequencing (NGS) techniques [5] and compared the microbial diversity with ground control samples [4]. The authors confirmed the anticipated composition of the ISS microbiota, as representing a typical human-associated community [4]. This observation appears logical due to the tremendous impact of the human microbiota on the environment (e.g., the human body spreads 10^6^ bacteria per hour through breathing [11]) and the severe restriction of other potential microbe sources. The major bacterial phyla detected by NGS-based methods were Firmicutes, Actinobacteria, and Proteobacteria [4], with the dominant genera *Corynebacterium* and *Propionibacterium*, representing typical human skin-associated microorganisms [12]. Besides NGS, cultivation-based approaches were also applied, which resulted in a high number of *Bacillus* and *Staphylococcus* isolates [4].

Standardized monitoring of surface and air samples on board the ISS as well as more detailed post-flight investigations have been and are currently being conducted (e.g., NASA’s Microbial Observatory Project). Another recent project, Merccuri (Microbial Ecology Research Combining Citizen and University Researchers on the ISS), studied 48 bacterial strains that were transferred from Earth environments to the ISS [3]. For most of the cultures, the researchers found no significant change with respect to growth rate during the few days incubation time, except for *Bacillus safensis*, which grew 60% better in space than on Earth [3]. However, selective pressures (desiccation, radiation, chemical, and physical stresses) on board the ISS could cause an adaptation of the indigenous microbiota towards ISS conditions during a longer time frame [13, 14].

The questions concerning a possible adaptation of microorganisms towards ISS stresses are addressed within an ESA flight project originally named “ARBEX” (Archaeal and Bacterial Extremophiles on board the ISS) now “Extremophiles” [2], which aims to analyze the adaptation processes of moderate and extremotolerant Bacteria and Archaea on the ISS. Thus far, Archaea have not been found in samples from the ISS [4] but have frequently been detected in human-associated environments and clean rooms [15], as they are integral part of the human skin and gut microbiota [16, 17] and can therefore also be expected on board the ISS.

The ARBEX project focuses on the hardiest microorganisms inhabiting the ISS and assessing their diversity and capabilities to resist certain stresses. For this study, we specifically selected dust samples from the Russian modules that were obtained 8–12 years ago and stored since then under dried and sealed conditions on Earth. Targeting long-time survivors and spore-forming microorganisms, we consequently assessed the cultivable microbial community of these samples, in order to obtain model microbial strains that could be utilized in analyzing specific adaptation towards environmental stresses, such as desiccation and lack of nutrients. We analyzed these microorganisms with respect to their resistance to thermal stress and clinically relevant antibiotics. In addition, we assessed the bacterial and archaeal communities from the stored dust samples via molecular methods (next-generation sequencing, NGS) and compared our new data with the previously derived information from the ISS microbiome [4].

## Methods

### Origin of samples

Extracts of different ISS samples obtained from the Russian Service Module of the ISS were provided by T. Alekhova and her team. Dust samples were retrieved during ISS-expedition 9 in October 2004 and during ISS-expedition 16 in April 2008: sample 1: “Dust filter-1 (2004),” dust filter of ventilation system (internal abbreviation: RISS1); sample 2: “Dust filter-2 (2004),” dust filter of ventilation system (internal abbreviation: RISS4); sample 3: “Dust collector (2004),” from vacuum cleaner (internal abbreviation: RISS5); sample 4: “Dust filter (2008),” dust filter of ventilation system (internal abbreviation: RISS3); sample 5: “Dust collector (2008),” from vacuum cleaner (internal abbreviation: RISS2). During the entire time after retrieval, the vacuum cleaner bags and dust filters were stored sealed (never opened since sampling on the ISS), under dry conditions at ambient temperature. Culture controls done from an unused, sterile dust collector and dust filter were negative.

### Dust extraction protocol

For extraction, a 0.9% *w*/*v* NaCl solution was prepared using heat-treated NaCl (24 h, 250 °C, in order to degrade remnants of contaminating DNA) and autoclaved PCR-grade water (LiChrosolv, Merck Millipore). Three 5–10 cm^2^ pieces of fabric were aseptically cut out of the vacuum cleaner bags and dust filters and submerged in 15 ml 0.9% DNA-free NaCl solution.

The fabric pieces in solution were then vortexed for 10 s, manually shaken for 15 s, ultra-sonicated at 40 kHz for 2 min, and finally vortexed for 10 s to detach the dust from the fabric. The fabric was aseptically removed from the solution, and resulting suspension and solid fabric pieces were used for cultivation and molecular analyses.

### Cultivation assays

The solid fabric was placed on aerobic R2A plates (pH 7; BDH Prolabo®), whereas the homogeneous dust suspensions were used to inoculate different culture media in duplicates. The media used are given in Table 1. Liquid media were inoculated once with 500 μl and once with 250 μl of the dust suspension, and solid media were inoculated once with 200 μl and once with 100 μl of the dust suspension. Since the focus was to isolate bacterial and archaeal isolates, all aerobic media were supplemented with a final concentration of 50 μg/ml nystatin to suppress fungal growth.

**Table 1:** List of used media and conditions

Medium	Phase	pH	Incubation temperature	Gasphase	Abbreviation	Target organisms	Medium reference
R2A agar pH 5	Solid	5	30 °C	Aerobic (ambient)	pH 5	Acidophiles	–
R2A agar pH 9	Solid	9	30 °C	Aerobic (ambient)	pH 9	Alkaliphiles	–
R2A agar pH 7	Solid	7	30 °C	Aerobic (ambient)	pH 7F	Heterotrophs	–
RAVAN pH 7 for oligotrophs	Solid	7	30 °C	Aerobic (ambient)	RAV	Oligotrophs	[72] (modified^a^)
DSMZ_Medium97 for halophiles	Liquid	7.5	40 °C	Aerobic (ambient)	Halo	Halophiles	DSM 97 (www.dsmz.de)
R2A pH 7 liquid	Liquid	7	30 °C	N_2_	pH 7an	Anaerobes	–
Medium for methanogens	Liquid	7	40 °C	H_2_CO_2_ (80:20)	MS	Methanogens	[73]
MS supplemented with 0.1% yeast extract and 0.1% acetate	Liquid	7	40 °C	H_2_CO_2_ (80:20)	MS_sup	Methanogens	–
Archaea-supporting liquid medium	Liquid	7	30 °C	N_2_	ASM	Archaea	[74]^b^
ASM supplemented with 0.1% yeast extract and 0.1% acetate	Liquid	7	30 °C	N_2_	ASM_sup	Archaea	–
Autotrophic all-rounder liquid medium	Liquid	7	30 °C	N_2_CO_2_ (80:20)	AAM	Autotrophs	[74]^b^
Autotrophic homoacetogen liquid medium	Liquid	7.5	30 °C	H_2_CO_2_ (80:20)	AHM	Autotrophs	[74]^b^

^a^1:100 diluted, final concentration of 50 mg/l sodium pyruvate instead of 20 mg/l pyruvic acid

^b^Without addition of antibiotics

Pure cultures were obtained via repeated dilution series in liquid medium and purification streaks on solid media. Positive enrichments of medium pH 7 were transferred to anaerobic R2A plates and then purified by purification streaks.

### Identification of isolates

Partial 16S rRNA genes of the isolates were amplified using the primers 9bF (5′-GRGTTTGATCCTGGCTCAG-3′) and 1406uR (5′-ACGGGCGGTGTGTRCAA-3′), applying the following cycling conditions: initial denaturation at 95 °C for 2 min, followed by 10 cycles of denaturing at 96 °C for 30 s, annealing at 60 °C for 30 s and elongation at 72 °C for 60 s, followed by another 25 cycles of denaturing at 94 °C for 30 s, annealing at 60 °C for 30 s and elongation at 72 °C for 60 s, and a final elongation step at 72 °C for 10 min [18]. The template was either a small fraction of a picked colony in a colony-PCR assay or 5–20 ng of DNA purified from culture via the peqGOLD Bacterial DNA Kit (peqlab, Germany). The 16S rRNA gene amplicons were Sanger-sequenced (Eurofins, Germany), and the obtained sequences were classified using the EzTaxon identification service at http://www.ezbiocloud.net/ [19].

The ITS sequence of one fungal isolate was sequenced using the primers ITS1F(5′-CTTGGTCATTTAGAGGAAGTAA-3′) and ITS4(5′-TCCTCCGCTTATTGATATGC-3′) and following cycling conditions: initial denaturation at 95 °C for 10 min, followed by 35 cycles of denaturing at 94 °C for 60 s, annealing at 51 °C for 60 s, elongation at 72 °C for 60 s, and a final elongation step at 72 °C for 8 min. The amplicons were Sanger-sequenced (Eurofins, Germany), and the obtained sequence was classified using the EzFungi identification.

### DNA extraction of original samples and incubation experiment

After aliquots were removed for cultivation assays, the remaining dust suspension was centrifuged at 16,000 *g* to concentrate the remaining dust particles and microorganisms, which were then re-suspended in three aliquots of 0.5 ml of the supernatant. One aliquot was directly frozen at −80 °C, and one was treated with an end concentration of 50 μM propidium monoazide (PMA), to block free DNA of dead cells from downstream applications [20] before freezing.

One aliquot was mixed with 0.5 ml of pre-warmed 30 °C LB medium and incubated at 30 °C for 1.5 h prior to direct DNA extraction with the aim of increasing the biomass and possibly triggering the germination of spores, which have been reported to resist state-of-the-art DNA extraction methods [5]. DNA was extracted using the modified XS-Buffer method as described previously [15]. DNA concentrations were determined using Qubit, and DNA was afterwards subjected to PCR.

### Molecular microbial diversity analysis using next-generation sequencing methods

To investigate the detectable molecular diversity, we used a “universal” and an Archaea-targeting approach. The 16S rRNA gene amplicons for the universal approach were amplified using Illumina-tagged primers F515 (5′-TCGTCGGCAGCGTCAGATGTGTATAAGAGACAG GTGCCAGCMGCCGCGGTAA-3′) and R806 (5′-GTCTCGTGGGCTCGGAGATGTGTATAAGAGACAG GGACTACHVGGGTWTCTAAT-3′) [21]. Archaeal amplicons were obtained via a nested approach [16]: First, a ~550 bp-long 16S rRNA gene amplicon was created via the primers Arch344F (5´-ACGGGGYGCAGCAGGCGCGA-3′) and Arch915R (5′-GTGCTCCCCCGCCAATTCCT-3′) [22, 23], and in a second PCR, the amplicons for Illumina sequencing were generated by the tagged primers S-D-Arch-0349-a-S-17 (5′-TCGTCGGCAGCGTCAGATGTGTATAAGAGACAG GYGCASCAGKCGMGAAW-3′) and S-D-Arch-0519-a-A-16 (5′-GTCTCGTGGGCTCGGAGATGTGTATAAGAGACAG TTACCGCGGCKGCTG-3) [24], using the purified product of the first PCR as template [16].

The cycling conditions for the universal approach were initial denaturation at 94 °C for 3 min, followed by 35 cycles of denaturing at 94 °C for 45 s, annealing at 60 °C for 60 s and elongation at 72 °C for 90 s, and a final elongation step at 72 °C for 10 min. For the first PCR of the nested archaeal approach, the cycling conditions were initial denaturation at 95 °C for 2 min, followed by 10 cycles of denaturing at 96 °C for 30 s, annealing at 60 °C for 30 s and elongation at 72 °C for 60 s, followed by another 15 cycles of denaturing at 94 °C for 30s, annealing at 60 °C for 30 s and elongation at 72 °C for 60 s, and a final elongation step at 72 °C for 10 min. For the second amplification the cycling conditions were initial denaturation at 95 °C for 5 min, followed by 25 cycles of denaturing at 95 °C for 40 s, annealing at 63 °C for 120 s and elongation at 72 °C for 60 s, and a final elongation step at 72 °C for 10 min.

Library preparation and sequencing were carried out at the Core Facility Molecular Biology at the Center for Medical Research at the Medical University Graz, Austria. In brief, DNA concentrations were normalized using a SequalPrep™ normalization plate (Invitrogen), and each sample was indexed with a unique barcode sequence (8 cycles index PCR). After pooling of the indexed samples, a gel cut was carried out to purify the products of the index PCR. Sequencing was done using the Illumina MiSeq device and MS-102-3003 MiSeq® Reagent Kit v3-600cycles (2 × 251 cycles).

### Antimicrobial susceptibility tests

Nineteen of the isolates were selected for antimicrobial susceptibility testing and heat-shocks based on their phylogeny and on differences in phenotypical appearance. Antimicrobial susceptibility testing for selected, clinically relevant antibiotics (Table 2) was performed using Etest® reagent strips (Biomérieux, Germany) according to instructions of the manufacturer. Since there were no species specific breakpoints available, MICs were interpreted according to EUCAST guideline table “PK/PD (Non-species related) breakpoints” [25].

**Table 2:** Antibiotics used in this experiment (additional information from [75] and [76]

Antibiotic substance	Type	Mechanism of action	Target group	Concentrations applied (μg/ml)
Amoxicillin/clavulanic acid	β-Lactam antibiotic (penicillin) and β-lactamase inhibitor	Inhibits cell wall synthesis; bactericidal against growing bacteria	Gram+ and Gram− bacteria	0.016−256
Ampicillin	β-Lactam antibiotic penicillin	Inhibits cell wall synthesis; bactericidal against growing bacteria	Gram+ and Gram− bacteria	0.016−256
Cefotaxime	β-Lactam antibiotic; cephalosporin	Inhibits cell wall synthesis; bactericidal against growing bacteria	Gram+ and Gram− bacteria	0.016−256
Ceftriaxone	β-Lactam antibiotic; cephalosporin	Inhibits cell wall synthesis; bactericidal against growing bacteria	Gram+ and Gram− bacteria	0.016−256
Ciprofloxacin	Fluoroquinolone	Inhibits bacterial DNA gyrase; bactericidal	Gram+ and Gram− bacteria	0.002−32
Clarithromycin	Macrolide	Inhibits protein synthesis; bacteriostatic	Gram+ and Gram− bacteria	0.016−256
Clindamycin	Lincosamide	inhibits protein synthesis; bacteriostatic	Gram+ and anaerobic Gram− bacteria	0.016−256
Colistin	Polypeptide antibiotic; polymyxin	Attacks cell membrane; bactericidal	Gram− bacteria	0.016−256
Doxycycline	Polyketide antibiotic; tetracycline	inhibits protein synthesis; bacteriostatic;	Gram+ and Gram− bacteria	0.016−256
Gentamicin	Aminoglycoside	inhibits protein synthesis; bactericidal	Gram− and some Gram+ bacteria	0.016−256
Levofloxacin	Fluoroquinolone	Inhibits bacterial DNA gyrase; bactericidal	Gram+ and Gram− bacteria	0.002−32
Linezolid	Oxazolidinone	inhibits protein synthesis; bacteriostatic	Gram+ bacteria	0.016−256
Meropenem	β-Lactam antibiotic carbapenem	Inhibits cell wall synthesis; bactericidal against growing bacteria	Gram+ and Gram− bacteria	0.002−32
Moxifloxacin	Fluoroquinolone	Inhibits bacterial DNA gyrase; bactericidal;	Gram+ and Gram− bacteria	0.002−32
Penicillin G	β-Lactam antibiotic penicillin	Inhibits cell wall synthesis; bactericidal against growing bacteria	Gram+ bacteria	0.016−256
Trimethoprim/sulfamethoxazole	Dihydrofolate reductase inhibtor and sulfonamide	Inhibits tetrahydrofolate synthesis; bactericidal	Gram+ and Gram− bacteria	0.002−32
Vancomycin	Glycopeptide antibiotic	Inhibits cell wall synthesis; bactericidal against growing bacteria	Gram+ bacteria	0.016−256

In brief, overnight cultures (2–3-day cultures for slower-growing bacteria) were suspended in 0.9% saline to a turbidity of McFarland 0.5. One hundred microliters of this suspension was plated on standardized Müller-Hinton agar for antimicrobial susceptibility testing (Becton Dickinson). Etest® reagent strips were placed on the plates followed by aerobic incubation for 18 +/−2 h at 34 °C. Two strains were tested four times (in duplicate) on R2A pH 7 and incubated for 48 h at 34 °C, because of their inability to grow on Müller Hinton medium and their intrinsic slow growth.

### Heat-shock resistance test

The heat-shock test was carried out according to ESA standards [26]. This test is usually applied to quantify the bioburden of spacecraft, in order to identify hardy microorganisms that are potentially able to survive a spaceflight to other solar bodies. In brief, single colonies of 1–2-day old cultures were suspended in two test tubes containing 2.5 ml sterile phosphate buffered saline (PBS). As a control, one tube was kept at room temperature during the procedure. The other tube was placed in an 80 °C water bath and exposed for 15 min. Samples were immediately cooled down on ice for 5 min after incubation time.

The temperature was monitored using a separate pilot tube containing 2.5 ml PBS also in the water bath. Afterwards, 0.5 ml of the heat-shocked suspension and 0.5 ml of the room temperature suspension were plated and incubated at 30 °C for 3 days (72 h).

### Negative controls

Negative controls were performed thoroughly. Cultivation, extraction, PCR, and sequencing controls were analyzed in parallel with the processing of the samples. Cultivation controls were performed on two levels. First, the same, unused material (dust collector, dust filter material) as was used on the ISS was placed on cultivation medium. Secondly, extraction blanks were processed in parallel to the ISS material. All cultivation controls were negative (no growth of colonies). Extraction blanks used for DNA extraction, PCR, and sequencing revealed a low number of ribosomal sequence variants (RSVs, see below). These RSVs were removed from all datasets, if present in the samples (the removed RSVs are highlighted in Additional file 1: Table S1 and Additional file 2: Table S2).

### Bioinformatical analysis and data processing

Demultiplexed, paired reads were processed in R (version 3.2.2) using the R package DADA2 as described previously [27]. In brief, sequences were quality checked, filtered, and trimmed to a consistent length of ~270 bp (“universal” primer set) and ~140 bp (“archaea” primer set). The trimming and filtering were performed on paired reads with a maximum of two expected errors per read (maxEE = 2). Passed sequences were dereplicated and subjected to the DADA2 algorithm to identify indel-mutations and substitutions. After merging paired reads and chimera filtering, the sequences were assigned to a taxonomy using the RDP classifier and the SILVA v.14 trainset. The visualization was carried out using the R package phyloseq [28, 29], and metabolic pathways were predicted using the R package Tax4Fun [30]. Biostatistical analyses were performed using STAMP [31].

In contrast to previously described data processing pipelines such as QIIME [32] and mothur [33], the DADA2 output table was not produced based on a clustering step and thus no operational taxonomic units (OTUs) were generated. Each row in the DADA2 output table corresponds to a non-chimeric inferred sample sequence, each with a separate taxonomic classification (ribosomal sequence variants; RSVs) [27]. In addition, the merging step occurs after denoising, which increases accuracy.

In order to compare our results to the recently published microbial community of US American ISS HEPA filter particulates and vacuum cleaner bag components (ISS Debris) [4], the protocol was changed as follows: Checinska et al. could not merge the forward and reverse reads using the software mothur [33] and also the DADA2 approach, which we applied, did not result in a sufficient amount of merged sequences (data not shown). For the sake of comparability, we reanalyzed the datasets of Checinksa et al. 2015 containing dust samples (i.e., “ISS HEPA total,” “ISS HEPA viable,” “ISS Debris total,” and “ISS Debris total”) and our dataset in parallel, by using only high quality forward reads (length ~130 bp, quality score: >30). This approach was in congruence with the data processing as described in Checinska et al. 2015 [4].

For phylogenetic tree construction, the sequence dataset was aligned and processed in MEGA 6 [34]. Alignment was minimized and cropped to the core area, on which tree calculation (maximum likelihood) was based on. The obtained tree and the data were visualized using iTOL [35]. The Venn diagram was created using the online tool InteractiVenn [36].

### Data availability

Sequencing datasets as well as partial 16S rRNA gene sequences of bacterial isolates and ITS1 sequence of the fungal isolate were submitted to the European Nucleotide Archive and are publicly available. Sequencing datasets are assigned the study project number PRJEB14961, and samples are named according to internal abbreviations RISS1-5 as described in the chapter “Origin of samples.” The accession numbers assigned to the partial 16S rRNA gene sequences and ITS1 sequence are LT617056-LT617090.

## Results

The International Space Station is an extreme working and living environment. It is completely sealed off from the outside and thus exhibits a unique combination of chemical and physical parameters that act on all abiotic and biotic matter. To date, the effect on the human body or on the microbial community therein is only sparsely studied. However, we hypothesize that the microbial community thriving and surviving inside of the International Space Station becomes adapted to desiccation and other stresses. For this reason, we analyzed 8–12-year-old dust samples from Russian ISS modules with respect to the cultivable portion of the microorganisms and the microbial community composition of these old samples. The retrieved microbial isolates were analyzed with respect to their resistance towards heat-shocks and antibiotics. Overall, our data were compared to recently obtained results from present day ISS samples [4].

### Numerous bacteria survive long-term archiving of International Space Station dust samples

We applied a variety of different culture media to retrieve microbial isolates from ISS samples. The culture media supported slightly acidotolerant, alkalitolerant, and oligotrophic microorganisms, respectively, but also provided growth conditions for autotrophs and anaerobic microbes. Overall, 85 bacterial isolates were obtained (Table 3) which could be assigned to eight genera. In spite of the nystatin applied to prevent fungal growth, we also obtained one single fungal isolate from “Dust filter-2 (2004)” on R2A pH 9. The fungal isolate was classified according to its internal transcribed spacer (ITS) sequence as *Ulocladium botrytis*.

**Table 3:** Number of bacterial isolates obtained on different cultivation media

	Number of microbial isolates obtained on culture media							
Sample origin	R2A pH 5	R2A pH 9	R2A pH 7F	R2A pH 7 anox	DSM97 “Halo”	MS_sup	ASM_sup	Total
1: Dust filter-1 (2004)	5	0	3	0	0	0	0	8
2: Dust filter-2 (2004)	5	0	2	0	0	0	0	7
3: Dust collector (2004)	10	7	3	3	0	2	1	26
4: Dust filter (2008)	5	1	4	0	0	0	0	10
5: Dust collector (2008)	14	11	3	3	1	2	0	34
Total	39	19	15	6	1	4	1	85

The highest percentage of isolates (46%) was obtained from R2A agar with pH 5, thus indicating a preference of slightly acidic growth conditions compared to pH 9 (22%) and pH 7 (18%). Additional growth was observed at pH 7 under anoxic conditions, whereas only a few isolates were obtained under high-salt concentrations (18% NaCl, *Salinibacillus aidingensis*), or in liquid MS_sup or ASM_sup medium. Only seven isolates were obtained from the “Dust filter-2 (2004)” sample, and the highest amount of isolates was obtained from the “Dust collector (2008)” sample.

**Fig. 1:**
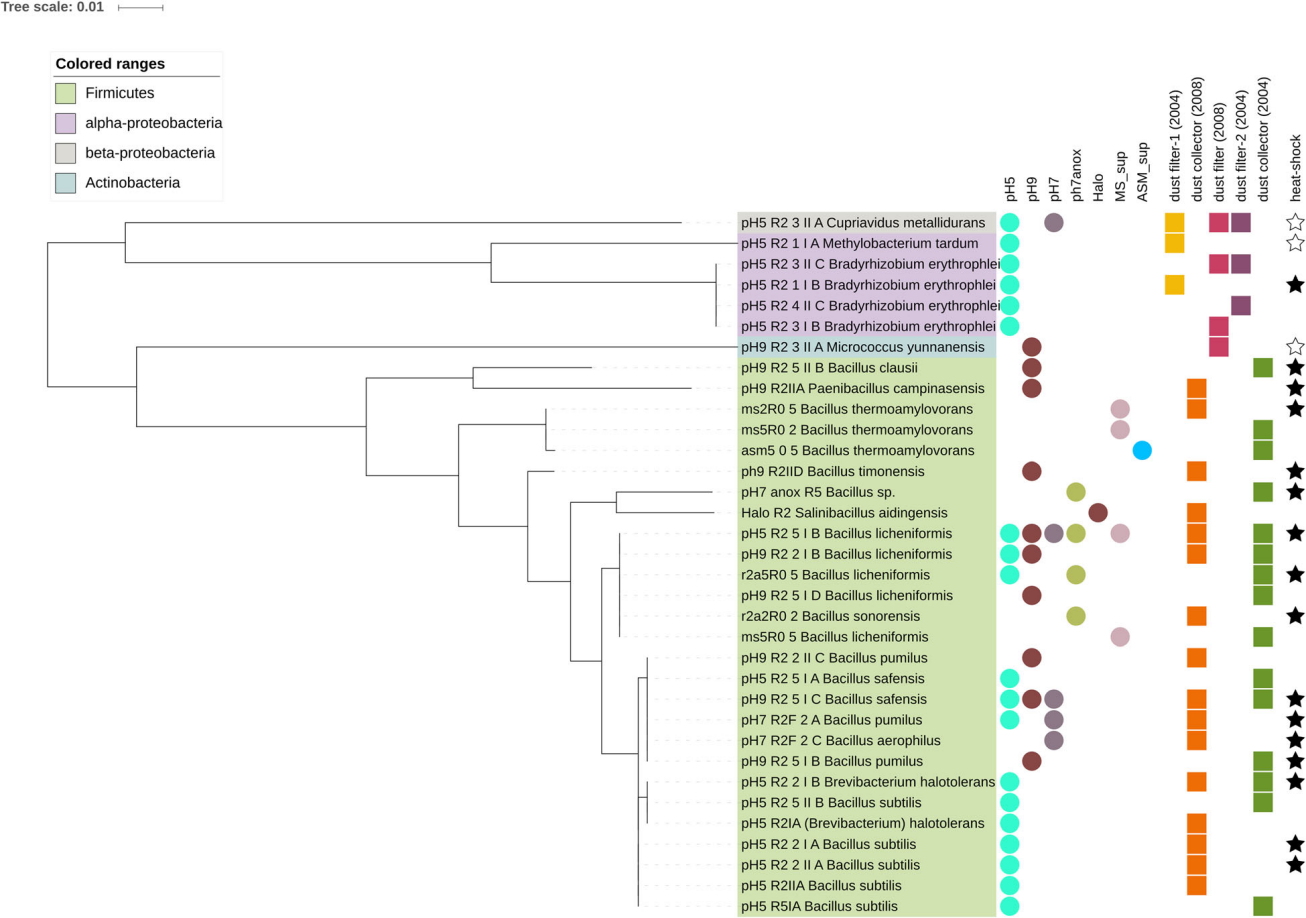
Maximum-likelihood tree based on the unique 16S rRNA gene sequences of the ISS isolates. *Circles* indicate the medium they were cultivated in. *Squares* refer to the sample origin. *Stars* indicate the heat-shock resistance of the isolates (*filled star*: survived heat-shock at 80 °C for 15 min; *empty star*: did not survive heat-shock; others were not tested). Tree was constructed using MEGA6 [34] and displayed by iToL [35]

Overall, 34 bacterial isolates were found to be unique with respect to their 16S rRNA gene, which are displayed according to their phylogeny, preferred culture medium and sample origin in Fig. 1.

Notably, isolates from dust filter samples were non-spore-forming Proteobacteria (*Cupriavidus*, *Methylobacterium*, *Bradyrhizobium*) and Actinobacteria (*Micrococcus*), whereas spore-forming species (Firmicutes, mainly *Bacillus* representatives) were isolated from dust collector samples only. These 34 different bacterial strains were assigned to 17 different species, which are listed in Additional file 3: Table S3, together with isolates obtained by Checinska et al. 2015 [4].

### ISS isolates were found to be resistant against desiccation, heat-shock, and some common clinically applied antibiotics

All isolates obtained had been stored in dust/dust filters for at least 8 years before the cultivation experiments were performed. Since they were stored under dry conditions, all cultivated strains can be assumed to be desiccation resistant.

For the heat-shock resistance and antibiotic susceptibility tests, 19 representative isolates were selected from our pool. Following the NASA and ESA guidelines for bioburden detection in clean rooms and on spacecraft [26], cultures were exposed to a heat-shock (15 min, 80 °C). This heat-shock is currently used by the named space agencies in order to determine the resistance to environmental stresses and to analyze whether microorganisms are possible candidates to survive space flight for planetary protection considerations. Sixteen out of 19 isolates survived the heat-shock treatment, as indicated in Fig. 1 (filled stars). All spore-forming microorganisms of the genus *Bacillus* and one *Paenibacillus* were able to survive the treatment, whereas *Methylobacterium*, *Cupriavidus*, and *Micrococcus* could not be re-grown afterwards. Notably, a few cells (three colonies instead of bacterial lawn as observed for spore-formers and positive control) of *Bradyrhizobium*, although representing a non-spore-forming Alphaproteobacterium, survived the heat-shock.

Seventeen clinically relevant antibiotics (see Table 2) were selected for susceptibility testing of the 19 selected microbial isolates. When applicable, the antimicrobial resistance was assessed using the most recent update of the EUCAST expert rules ([37]; www.eucast.org; Breakpoint Table for bacteria v6.0, January 2016). The results of the antibiotics-resistance tests are summarized in Table 4 (see also Additional file 4: Figure S1).

**Table 4:** Minimal inhibitory concentrations for the tested isolates

Antibiotics	Isolate	pH 5_R2_1_I_A *Methylobacterium tardum*	pH 5_R2_1_I_B *Bradyrhizobium erythrophlei*	pH 5_R2_1_II_A *Cupriavidus metallidurans*	pH 5_R2_2_I_B *Brevibacterium halotolerans*	pH 5_R2_2_I_A *Bacillus subtilis*	pH 5_R2_2_II_A *Bacillus subtilis*	pH 5_R2_5_I_B *Bacillus licheniformis*	pH 7_R2F_2_A *Bacillus pumilus*	pH 7_R2F_2_C *Bacillus aerophilus*	pH 9_R2_3_II_A *Micrococcus yunnanensis*	pH 9_R2_5_I_B *Bacillus pumilus*	pH 9_R2_5_II_B *Bacillus clausii*	pH 9_R2_5_I_C *Bacillus safensis*	r2a5R0,2 *Bacillus licheniformis*	r2a5R0,5 *Bacillus licheniformis*	ms2R0,5 Bacillus *thermoamylovorans*	pH7_anox_R2 *Bacillus licheniformis*	pH9_R2IIA *Paenibacillus campinasensis*	pH9_R2IID *Bacillus timonensis*
Max. concentration: 256 μg/ml																				
Amoxicillin/clavulanic acid		2	**256** ^a^	*1.5*	2	3	*0.5*	7	3	*0.25*	*0.75*	2	*0.19*	3	**16**	**12**	*0.19*	3	**256** ^a^	2
Cefotaxime		**12**	**256** ^a^	1	2	**8**	**16**	**64**	**256** ^a^	**128**	*0.75*	**16**	**32**	**256** ^a^	**24**	**32**	**32**	**256** ^**a**^	**12**	**32**
Ceftriaxone		1.5	**256** ^a^	*0.5*	1.5	*0.38*	**12**	**256** ^a^	**32**	**16**	*0.19*	**4**	**12**	**24**	**24**	**12**	**8**	**64**	**12**	**48**
Linezolid		**256** ^a^	**256** ^a^	**256** ^a^	*1*	*1*	*0.75*	*1*	2	*1.5*	*1*	*0.75*	*1*	2	*1.5*	*1*	*1*	2	**6**	*1*
Penicillin G		**3**	**256** ^a^	**3**	**8**	0.75	0.5	**256** ^a^	0.38	*0.064*	*0.094*	**256** ^a^	**24**	0.25	**24**	**6**	**4**	0.75	**24**	1
Ampicillin		**256** ^**a**^	**256** ^a^	*1.5*	2	3	**16**	**256** ^a^	2	*0.25*	*0.5*	*1.5*	**24**	2	**24**	8	2	3	**256** ^a^	2
Colistin		256*	256^a^	24	32	32	64	64	48	12	2	32	3	24	32	32	64	24	48	16
Clarithromycin		2	256^a^	2	0.094	0.064	0.064	0.38	0.125	0.064	4	0.064	256^a^	0.5	0.064	256^a^	256^a^	0.032	256^a^	0.032
Clindamycin		256*	256^a^	256^a^	0.5	0.75	0.5	12	3	2	0.25	24	256^a^	4	4	8	1	4	256^a^	2
Doxycycline		1.5	256^a^	0.064	0.047	0.094	0.19	0.25	0.19	0.094	0.25	0.064	0.25	0.125	0.125	0.25	0.75	0.38	0.125	0.19
Gentamicin		8	96	32	0.094	0.094	0.75	0.38	0.125	0.064	0.38	0.5	0.064	0.094	0.19	0.75	0.125	0.064	0.75	0.064
Vancomycin		256^a^	256^a^	0.75	256^a^	1	2	3	1.5	1	0.19	2	0.75	0.75	1	8	2	2	4	1.5
Max. concentration: 32 μg/ml																				
Ciprofloxacin		**6**	**32** ^a^	*0.016*	*0.047*	*0.064*	*0.064*	*0.125*	*0.19*	*0.094*	0.75	*0.032*	0.75	*0.094*	*0.094*	*0.047*	*0.19*	*0.125*	*0.125*	*0.125*
Levofloxacin		**3**	**32** ^a^	*0.047*	*0.094*	*0.125*	*0.094*	*0.19*	*0.25*	*0.125*	1.5	*0.094*	2	*0.125*	*0.094*	*0.064*	*0.38*	*0.125*	*0.094*	*0.094*
Meropenem		**32** ^a^	**32** ^a^	*0.125*	*0.064*	*0.19*	*0.094*	*0.19*	*0.25*	*0.094*	*0.47*	*0.094*	*1*	*0.25*	**19**	**19**	*0.38*	*0.38*	3	*0.38*
Moxifloxacin		0.25	**32** ^a^	*0.023*	*0.016*	*0.047*	*0.023*	*0.032*	0.640	*0.016*	*0.38*	*0.008*	0.5	*0.023*	**16**	*0.012*	*0.032*	*0.025*	*0.047*	*0.023*
Trimethoprim/sulfamethoxazole		32^a^	32^a^	0.064	0.125	0.19	0.19	0.19	0.004	0.004	0.5	0.032	0.047	0.094	0.032	0.032	0.094	0.006	0.75	0.004

*Italic underlined*: sensitive according to non-species related breakpoints of EUCAST; *Bold*: resistant according to non-species related breakpoints of EUCAST; Slow growing *M. tardum* and *B. erythrophlei* were tested on R2A medium with 2 days incubation time since they did not grow at all on MH medium

^a^Grew at the tested maximum concentration of the respective antibiotic; concentrations given in: μg/ml

All strains were transferred to Müller-Hinton agar, and antibiotic tests were performed using this medium, with the exception of two strains: *Methylobacterium tardum* and *Bradyrhizobium erythrophlei*, as they resisted growing under these conditions. For these two strains, we used R2A and an incubation time of 48 h. Both strains revealed, under adapted conditions, robust resistances against numerous antibiotics. All strains revealed resistance against at least one antibiotic compound above the non-species specific EUCAST threshold, except *Micrococcus yunnanensis*.

We tested six different β-lactam antibiotics of which the cephalosporins cefotaxime and ceftriaxone as well as penicillin G were found to be most ineffective against the ISS isolates, since almost all microbial strains exceeded their resistance breakpoints or at least the intermediate breakpoints (see Table 4 and Additional file 4: Figure S1A).

### Molecular, NGS-based analysis revealed the presence of a broad bacterial and archaeal diversity

Aliquots of the same samples that were used for the cultivation approach were subjected to molecular analyses. We followed three different approaches. (A) Samples were processed untreated, (B) Samples were exposed to liquid growth medium (LB) for 1.5 h (30 °C) in order to increase biomaterial and trigger spore germination (incubated samples), (C) Samples were treated with propidium monoazide (PMA) to mask background DNA from disrupted cells [38]. PMA-treated samples (C) did not reveal any signals after DNA extraction and PCR, using “universal” and archaea-targeting primer sets, although cultivation from these samples was successful. However, all samples that underwent the incubation treatment (B) resulted in reasonable PCR product yields. Untreated samples (A) resulted in positive archaeal amplicon generation for four out of five samples, namely “dust filter-1 (2004),” “dust collector (2004),” “dust filter (2008),” and “dust collector (2008)”; three out of five samples resulted in positive universal amplicon generation (“dust filter-1 (2004),” “dust filter (2008),” “dust collector (2008)”).

Universal and archaeal amplicons were subjected to next-generation sequencing (Illumina MiSeq). Raw reads were processed using DADA2. It should be noted that DADA2 does not perform a clustering step, thus does not produce operational taxonomic units (OTUs). Each sequence obtained corresponds to a unique taxonomic classification (ribosomal sequence variant; RSV).

In total, 203,667 high quality sequence counts were obtained of the four positive archaeal approaches (length >140 bp), representing nine different RSVs. Among the four samples, the “dust collector (2004)” yielded the highest number of sequence counts (102,782). The “dust filter-1 (2004)” sample and the “dust collector (2008)” sample resulted in 71,203 and 29,600 archaeal sequence counts, respectively, whereas the lowest number was observed in the “dust filter (2008)” sample (82 sequence counts). As a consequence, the “dust filter (2008)” sample revealed the lowest richness, the lowest Shannon Index, and the lowest InvSimpson Index (Additional file 4: Figure S2). The highest archaeal richness was observed in the “dust filter-1 (2004)” sample (8 RSVs).

**Fig. 2:**
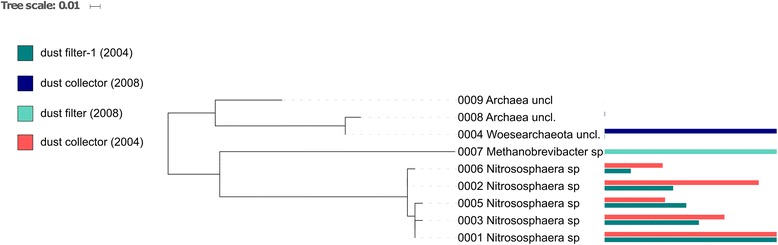
Archaeal maximum-likelihood tree: detected taxa and their abundance in different samples of ISS

**Fig. 3:**
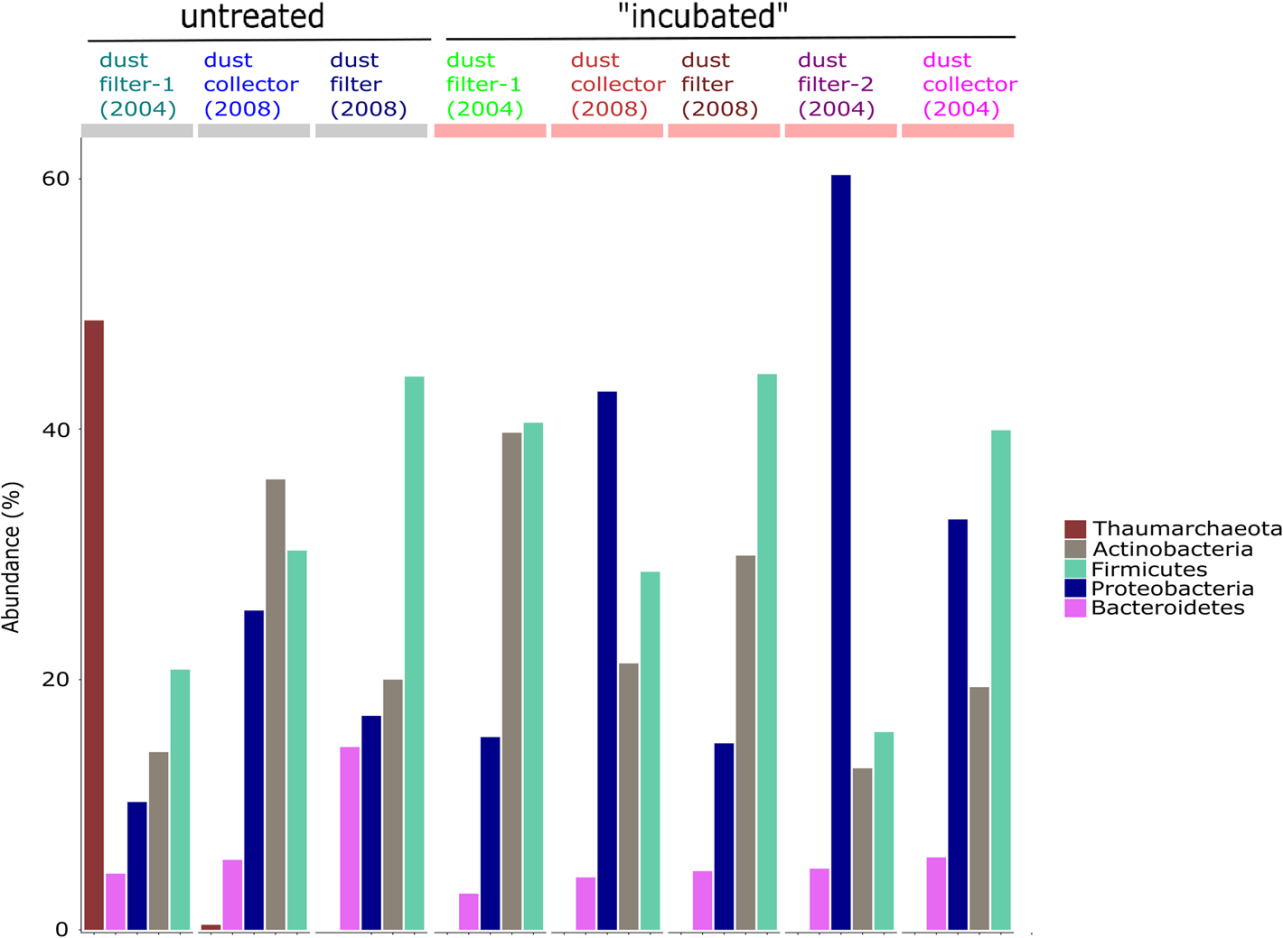
Taxonomic profiles of the microbial communities from Russian ISS samples at phylum level. The five most abundant phyla are depicted. We discriminate between untreated and incubated samples. Total counts are given in % (“Abundance”)

Overall, sequences assigned to Thaumarchaeota (*Nitrososphaera* sp.), Euryarchaeota (*Methanobrevibacter* sp.), and Woesearchaeota were found in the ISS samples (Fig. 2). *Methanobrevibacter* sequences could be detected in a very low abundance in “dust filter-1 (2004)” and also in “dust filter (2008),” where all obtained 82 sequence reads belonged to the genus *Methanobrevibacter* (see also Additional file 5: Table S4). Unclassified Woesearchaeota signatures were found in “dust filter-1 (2004)” at very low abundance (<0.1% of sample) and in “dust collector (2008)” with a very high abundance (>99.9% of sample; 14.5% of all archaeal sequence counts). *Nitrososphaera* signatures (Thaumarchaeota) were detected in two samples in a high abundance (“dust filter-1 (2004)” and “dust collector (2004)”), but were not observed in other samples. Furthermore, thaumarchaeal signatures were also detected in sequence data derived from amplicons produced with the universal primer pair. In particular, they were detected in untreated samples of “dust filter-1 (2004),” in agreement with the archaea-targeting approach mentioned above. 48.5% of the universal 16S rRNA gene sequences derived from this sample were assigned to Thaumarchaeota, soil crenarchaeotic group (SCG), with *Nitrososphaera* as the main genus. However, all other samples containing archaeal reads revealed only very low abundances (<1%). Those were mainly assigned to Euryarchaeota (Methanobacteria), represented by *Methanobrevibacter* (0.6% of incubated “dust filter-2 (2004)”), *Methanosphaera*, *Methanobacterium*, or not further classified members of the Woesearchaea (0.5% of “dust collector (2008)”; in congruence with the high amount of woesearchaeal reads obtained by the archaeal primer set for this sample). “Dust collector (2004),” the other sample with a high abundance of *Nitrososphaera* when sequenced with archaeal primers, did not deliver any sequences with the universal primer pair. In the incubated “dust filter-1 (2004)” and “dust collector (2004),” we could also not detect Thaumarchaeota with the universal primer set. In total, NGS based on the “suniversal” primer set generated 227,439 high-quality sequences (Additional file 6: Table S5). Sequences obtained by the universal primer approach were classified using the SILVA database [39], and community composition was summarized (see Fig. 3). In the following, we distinguish between untreated and incubated samples, referring to treatments A and B, respectively, as indicated above. In untreated samples, most signatures were assigned to phyla Thaumarchaeota (48.7%; “dust filter-1 (2004)”), Actinobacteria (36% in “dust collector (2008)”) and Firmicutes (44.2% in “dust filter (2008)” sample)). In incubated samples, the dominant phyla were Actinobacteria, Firmicutes, and Proteobacteria. Signatures of Bacteroidetes (lowest abundance in incubated “dust filter-1 2004” sample; highest abundance in untreated “dust filter 2008” sample) were also found in all samples. Further details are shown in Fig. 3.

At class level, the most abundant taxa were assigned to thaumarchaeal soil crenarchaeotic group (SCG), Actinobacteria, Bacilli, Gammaproteobacteria, and Betaproteobacteria. In incubated and untreated “dust filter (2008)” samples, there was no remarkable difference with respect to the microbial community composition. Five genera were identified whose abundance appeared significantly different in incubated and untreated samples (paired White test, *p* > 0.001, confidence >0.95, see Additional file 4: Figure S3): *Facklamia* (Lactobacillales; higher abundance in untreated sample), *Coprococcus_1* (Clostridiales; higher abundance in untreated sample), *Leuconostoc* (Bacilli; higher abundance in untreated sample); *Coproccocus*_3 (higher abundance in incubated sample), and an unclassified member of the family *Ruminococcaceae* (higher abundance in incubated sample).

**Fig. 4:**
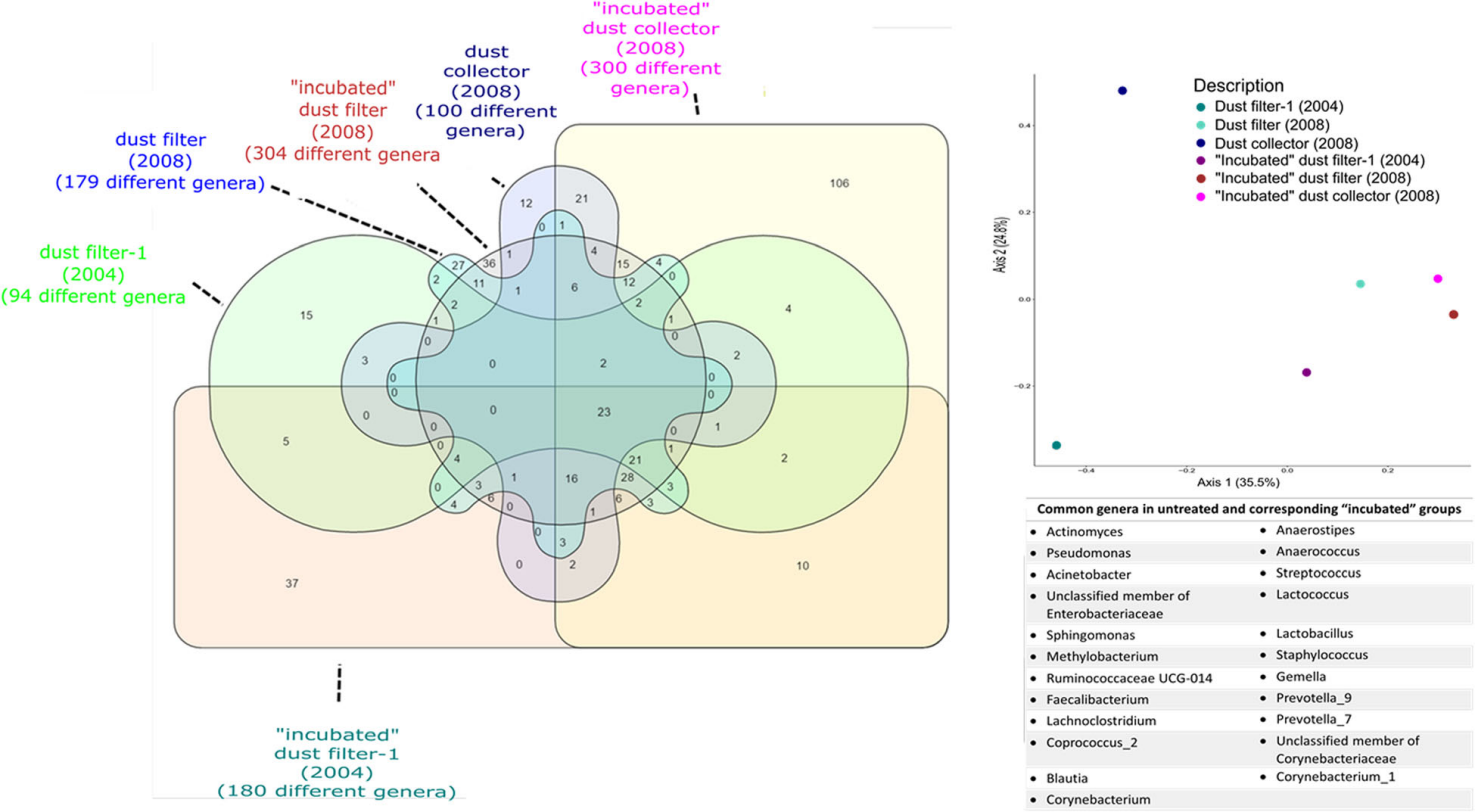
Venn diagram depicting common genera in untreated groups and their corresponding incubated counterparts. In total, signatures of 23 genera were common in all six samples. The PCoA plot on the right side is depicting the dissimilarity between incubated and untreated samples using the unweighted Bray-Curtis distance. No clear cluster pattern is visible between the two groups

In total, we could identify signatures of 23 microbial genera shared by untreated and corresponding incubated samples (Fig. 4). These taxa were mostly assigned to Actinobacteria (4), Clostridia (6), Bacilli (5), and Alpha/-Gammaproteobacteria (2 and 3, respectively). To compare community composition among samples, a beta-diversity matrix (i.e., Bray-Curtis distance (unweighted)) was computed and evaluated using principal coordinate analysis (PCoA, see Fig. 4). Untreated “dust filter (2008)” was found to reveal a similar microbial community composition as the incubated “dust filter (2008)” sample and the incubated “dust collector (2008)” sample. In contrast, the microbial community of the untreated “dust filter-1 (2004)” sample and the “dust collector (2008)” sample appeared to be distinct.

### Comparison between cultivation-based microbial diversity and molecular analysis emphasizes the need of cultivation

**Fig. 5:**
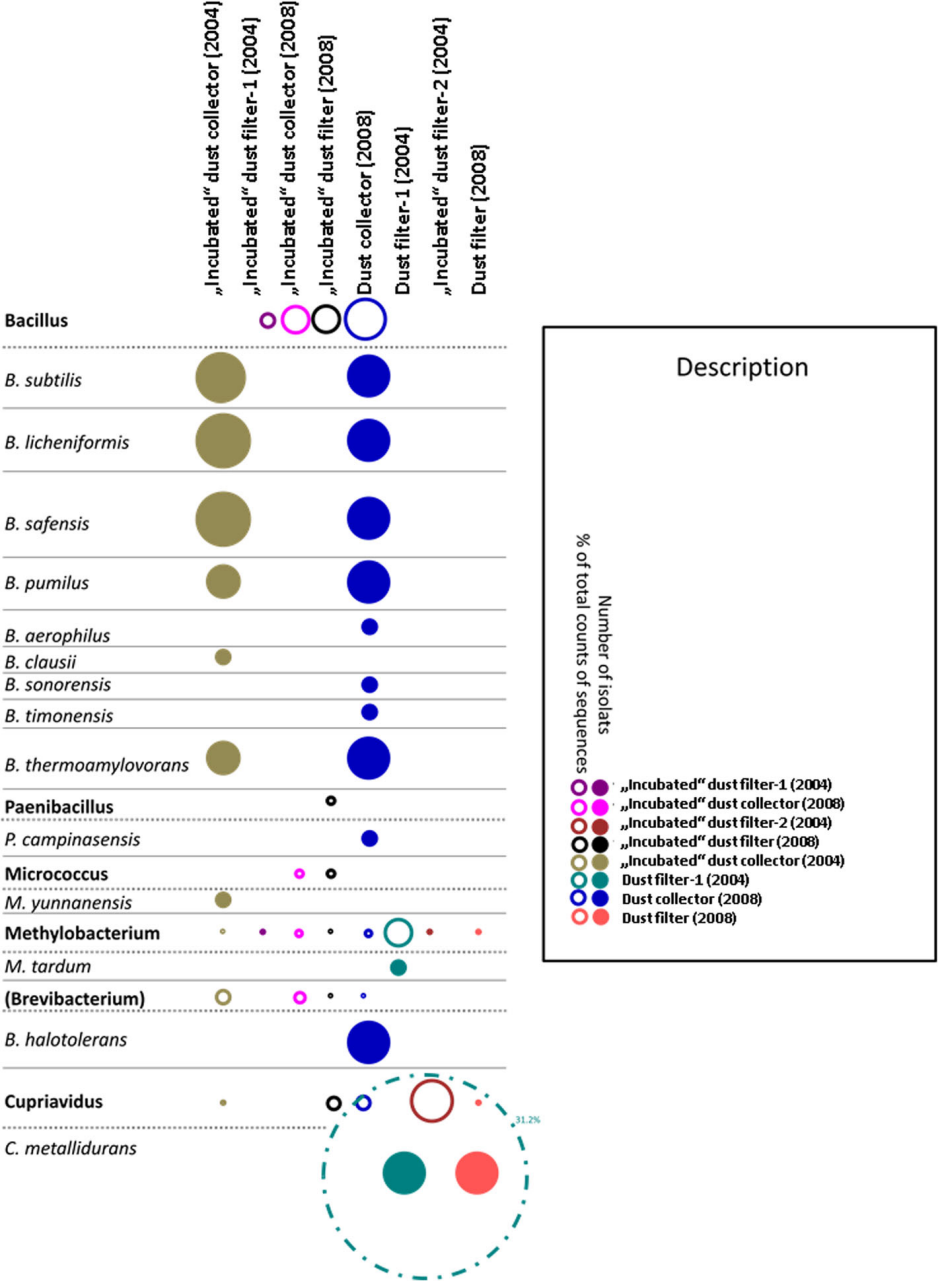
Comparison of the number of retrieved isolates with molecular data. *Donuts* indicate retrieved sequences on molecular level, *filled* circles are indicative for cultivated isolates. The larger the donuts/circles are, the more counts of sequences/isolates were obtained. Every sampling site has a different color (see legend). If no circle/donut appears, no sequences/isolates were obtained. It has to be noted, that no NGS data for the untreated “dust collector (2004)” could be retrieved; however, isolates from this sample could be cultivated

For the comparison of cultivation-based microbial diversity with the overall microbial diversity, we focused on the 34 unique isolates. The partial 16S rRNA gene sequences of individual, unique isolates were compared pairwise with all Illumina sequences belonging to the same genus. The sequences were considered to belong to the same species if they exceeded the similarity threshold of 99%. Almost all isolates could be retrieved in the sequencing results (see Fig. 5), but the isolates belonging to the genus *Bradyrhizobium* and *Salinibacillus* could not be detected in the sequence pool. In general, most isolates could be obtained from the “dust collector (2004)” and the “dust collector (2008),” whereas the highest counts of sequences could be obtained in the “dust filter-1 (2004)” (*Cupriviadus metallidurans*, 31.2% of all sequence reads). Remarkably, a high proportion of different *Bacillus* species could be isolated, in accordance with the total sequence count retrieved for all samples. However, half of the isolates were cultivated out of the “dust collector (2004),” where no sequences for *Bacillus* were detected at all. The non-conformity between sequencing data and isolated cultures is also true for species of *Paenibacillus* and *Micrococcus. Brevibaterium* signatures could be obtained in four of six sampling sites, and *Brevibacterium* isolates could be cultivated out of one sampling site. *Methylobacterium*, in contrast, was detected throughout all sampling sites but could be isolated out of only one sampling site.

### Differences between the microbial communities of archived ISS dust samples (this study) and more freshly retrieved samples [4]

Very recently, a dataset of microbial community composition of comparably fresh dust samples from the US American modules of the ISS has been published (ISS Hepa filter particulares, vacuum cleaner bag components of ISS (ISS Debris); retrieved 2011 and 2012; [4]). These data were retrieved and used for comparison by beta-diversity matrices (Bray-Curtis distance (unweighted); see also Additional file 7: Table S6).

We want to emphasize here, that for the sake of comparibility, we exclusively analyzed the forward reads of each study, since in contrast to our study, the reads in the study of Checinska et al. 2015 were not mergeable. However, the PCoA plot in Additional file 4: Figure S4 shows a clear clustering of the US American dust samples (US-ISS; [4]) and the Russian dust samples (RISS, this study), which indicates, despite insufficient possibility of data analysis, a certain dissimilarity of the microbial community in the two different ISS settings. In Table 5, we compared the data derived from Checinska et al. 2015 with our dataset, containing merged and further processed forward and reverse reads. A deeper look into the overall community shows a clear difference in the abundance of the dominant phyla. First, in contrast to the previous study, we were able to detect archaeal sequences, mainly classified as Thaumarchaea. Second, although both studies detected Actinobacteria, Firmicutes, and Proteobacteria as the dominant phyla in all samples, the mean abundance varied. The phylum Actinobacteria was observed to constitute ~64% of all samples in the US American ISS samples, whereas the older, archived Russian samples only harbored ~24%. In addition, the number of dominant genera was twofold reduced (mean US-ISS: 58 genera, mean RISS (Russian ISS samples): 28 genera). In contrast, the average abundance of Proteobacteria was increased ~8.7 times (mean values US-ISS: 3.255%, RISS: 27.4%); however, the number of genera was higher in US American ISS samples (mean number of genera RISS: 44.125 and mean number of genera US American ISS: 69.78). The amount of classified Firmicutes sequences and genera was comparable in both US American ISS and RISS samples (sequences mean: 25.78 and 28%, respectively, and mean number of genera 79 and 71.5, respectively).

**Table 5:** Comparison of microbial community composition of US American ISS dust samples [4] and Russian ISS dust samples (this study)

		US American ISS dust samples				Russian ISS dust samples							
		ISS HEPA total	ISS HEPA viable	ISS DEBRIS	ISS DEBRIS viable	Incubated dust filter-1 (2004)	Incubated dust collector (2008)	Incubated dust filter (2008)	Incubated dust filter-2 (2004)	Incubated dust collector (2004)	Dust filter-1 (2004)	Dust collector (2008)	Dust filter (2008)
	Total number of reads	553,176	587,569	1,148,047	1,116,419	24,382	61,571	49,956	7278	39,289	15,021	7599	22,343
	Percentages of sequences of all dominant phyla (Archaea and Bacteria)	90.92	99.65	92.35	98.26	95.6	92.9	89.2	89	92.1	93.9	92.2	81.3
	Percentages of sequences of dominant bacterial phyla (without Archaea)	90.92	99.65	92.35	98.26	95.6	92.9	89.2	89	92.1	45.2	91.8	81.3
Actinobacteria	Percentage of sequences	63.28	95.28	40.52	66.54	39.7	21.3	29.9	12.9	19.4	14.2	36.0	20.0
	Number of Genera	78	55	62	38	21	50	28	21	54	13	21	22
	Number of dominant genera^a^	16	7	28	16	8	16	11	2	15	5	4	5
Firmicutes	Percentage of sequences	24.83	3,97	45.67	28.48	40.5	28.6	44.4	15.8	39.9	20.8	30.3	44.2
	Number of Genera	118	67	100	31	89	105	92	23	99	41	39	84
	Number of dominant genera^a^	50	17	65	18	39	29	33	2	33	7	6	24
Proteobacteria	Percentage of sequences	2,81	0,41	6,16	3,24	15.4	43.0	14.9	60.3	32.8	10.2	25.5	17.1
	Number of Genera	95	65	89	30	35	82	43	20	88	19	26	40
	Number of dominant genera^a^	22	7	49	10	9	25	13	8	22	4	5	10
Thaumarchaea	Percentage of sequences	0.0	0.0	0.0	0.0	0.0	0.0	0.0	0.0	0.0	48.7	0.4	0.0
	Number of Genera	0	0	0	0	0	0	0	0	0	2	1	0
	Number of dominant genera^a^	0	0	0	0	0	0	0	0	0	1	0	0

^a^>100 sequence counts

### Prediction of resistance capacities

**Fig. 6:**
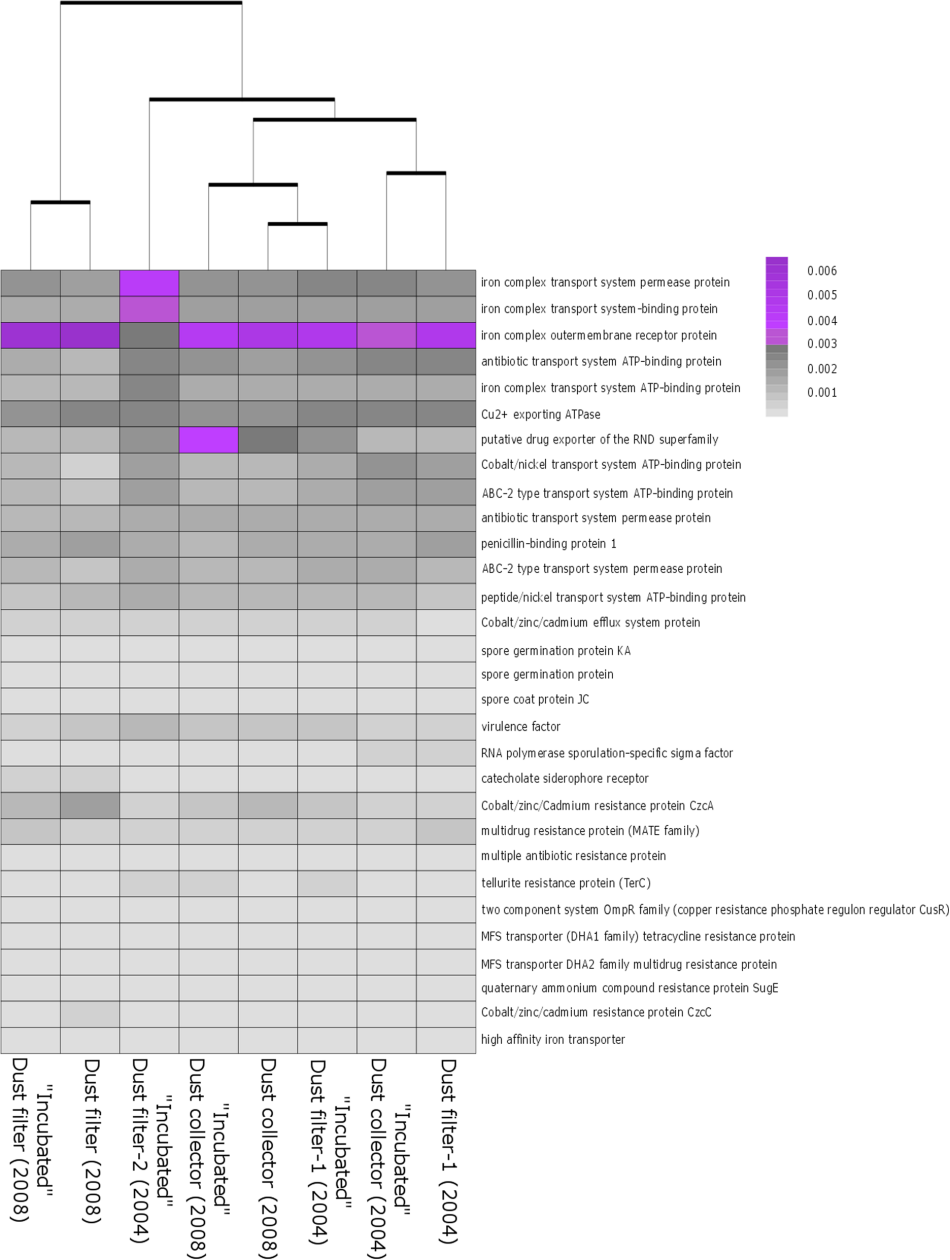
Hierarchical clustering of selected functional genes. The profiles clustered based on sampling site. The color scale reflects relative abundance of genes in % (*black*: low abundance, *violet*: high abundance)

Next to the phylogenetic diversity of microorganisms, we also wanted to retrieve information on which genes might be essential to their adaption to this extreme environment. It has to be emphasized that we did not apply a metagenomics approach to assess the entire set of functional genes but used the *in silico* tool Tax4Fun [30] to predict functional genes derived from our 16S rRNA amplicon dataset (universal primer set). In total, we obtained 6558 predicted single genes and 281 pathways (KEGG3 level). We focused on predicted genes/pathways responsible for antibiotic synthesis/resistance, transporters in general, resistance in general (e.g., resistance against metals or sporulation ability) and compared the individual relative abundances throughout all samples (Fig. 6). First, hierarchical clustering of selected functional genes resulted in two main clusters, consisting of the sample “Dust filter (2008)” with the incubated respective sample and the other samples forming the other cluster. There was no cluster pairing specifically incubated or untreated samples. The gene encoding for the iron complex “outer membrane receptor protein” was predicted to be highly abundant throughout all samples with the exception of the incubated sample “dust filter-2 (2004).” In general, genes encoding for resistances/adaptions were predicted to be equally distributed throughout all samples. These *in silico*-based predictions are not obligatorily reflecting the actual gene pool and need to be verified experimentally in future work.

## Discussion

In this communication, we retrieved novel information on the resistance capacities of resilient microorganisms derived from archived ISS dust samples. Our work allows deeper insight into the extremotolerant and adapted microbial community therein, revealing the presence of archaeal signatures as well as a robust microbial resistance machinery.

All 85 bacterial isolates survived for a prolonged time period of 8–12 years in desiccated dust. This implies a desiccation resistance achieved by different strategies, such as spore-forming capability or optimized DNA-repair mechanisms (Table 6). The origin of the bacterial isolates remains unknown, but many of them have already been detected in ISS or spacecraft-associated clean rooms or are typical human-associated microorganisms (Table 6.)

**Table 6:** Summary of proposed survival strategies of the isolates and their possible origin

Isolated microbial genus	Possible origin	Proposed survival strategy	References
*Bacillus*	HA, CR, ISS	Endospores	[77–83]
*Paenibacillus*	CR, ISS	Endospores	[78–81]
*Salinibacillus*	HA, ENV	Endospores	[40, 84]
*Micrococcus*	HA, CR, ISS, IA	Intrinsic desiccation resistance	[81, 82, 85–88]
*Cupriavidus*	CR, ISS	Various DNA-repair mechanisms; adapted to extreme, metal-rich, anthropogenic environments	[78, 89]
*Methylobacterium*	CR, ISS, IA	Intrinsic desiccation resistance	[5, 14, 20, 82, 90]
*Bradyrhizobium*	CR, ISS	Intrinsic desiccation resistance	[5, 14, 20, 81, 82, 91]

Legend: *HA* human-associated; *CR* reported in spacecraft assembly clean room(s) before; *ISS* reported in ISS before; *ENV* environmental; *IA* indoor air

It has to be pointed out that the origin of the halophile isolate *Salinibacillus* is very unclear. It has not been detected on board the ISS or in spacecraft assembly clean rooms before and is also not a typical human-associated bacterium. However, it has recently been detected in human stool, although the authors did suspect an erroneous classification [40].

Nineteen representative ISS isolates were tested for their ability to survive a heat-shock and their susceptibility to 17 clinically relevant antibiotics in vitro. As expected, all spore-forming isolates survived the heat-shock and non-spore-forming isolates did not, except for *B. erythrophlei*. A few colonies appeared after the heat-shock at 80 °C for 15 min. It has been reported that *Bradyrhizobium japonicum*, a close relative of *B. erythrophlei*, possesses multiple small heat-shock proteins that support survival of naturally occurring heat peaks of more than 40 °C [41]. However, since only a few survivors were found after the 80 °C treatment, one might assume that such heat-shock proteins, if present in *B. erythrophlei*, are not a reliable protection at these elevated temperatures.

The same strains were tested for their susceptibility against 17 clinically relevant antibiotics. It should be stressed that none of the isolates obtained was judged to be an opportunistic pathogen, and the antibiotic resistances remain without clinical relevance in this regard. However, the isolates revealed a remarkable pool of antibiotic resistance. Only the isolate *M. yunnanensis* showed no resistance towards all tested antibiotics. The most resistant isolate which could be evaluated according to the EUCAST standard (see the “Methods” section for more details) was *Paenibacillus campinasensis*, showing resistance against 8 of the 17 tested antibiotics.

During testing of the cephalosporines cefotaxime and ceftriaxone, we observed a high number of resistant isolates (16/19: cefotaxime and 14/19: ceftriaxone). However, most of the resistant isolates were *Bacillus* representatives, whereas our few Gram-negative isolates were mostly rated not resistant. Overall, *Bacillus* species appear to be rather resistant against cefotaxime and ceftriaxone, as reported before for e.g., *B. anthracis* [42–44].

However, of the two organisms that were tested under adapted conditions (due to no growth on Müller Hinton agar), *M. tardum* exceeded the non-species-related resistance breakpoints of 11 of the 17 antibiotics, whereas the *B. erythrophlei* isolate appeared to be even unaffected by almost all of the tested antibiotics except gentamicin. Of course these results have to be evaluated with extreme caution since not all of the EUCAST evaluation criteria could be met. *B. erythrophlei* was recently isolated from root nodules of Ironwood in south China and subsequently described as a new species [45]. Notably, *Bradyrhizobium* sp. (Accession number AY599676), now classified as *B. erythrophlei*, was also recently observed in propidium monoazide treated samples of spacecraft assembly facilities [20]. *Bradyrhizobium* signatures were reported in high abundance even in intensive care units and hospital biofilms [46, 47]. To date, the impact of *Bradyrhizobium* species (except for *B. enterica*) on human health remains elusive, but this genus has obviously a robust strategy for survival under stressful conditions. However, although *B. erythroplei* is not reported to be pathogenic, it could act as a reservoir for resistance genes on the ISS that might, under selection pressure, be passed on via horizontal gene transfer to infectious microorganisms.

A number of studies have been conducted on the reaction of bacteria to human spaceflight conditions, focusing on the changed pathogenic potential or resistance development [48]. For some microorganisms, an elevated virulence has been found, whereas others remained unaffected [48]. Recently, it has been shown for *Staphylococcus* species, that even a short-term stay in space can trigger the development of antimicrobial resistance [49]. In addition, decreased susceptibility of microbes to antibiotics under space-flight conditions have been reported [49]. Notably, bacterial infections ocurring during human space-flight on Mir or spaceshuttle have been observed earlier, such as infections of the urinary tract, upper respiratory tract, and subcutaneous tissue, as well as an increased reactivation of latent viral infections due to the deterioration of the astronaut´s immune system [49, 50].

Of the antibiotics we tested in this study, amoxicillin (without clavulanic acid), ceftriaxone, ciprofloxacin, clindamycin, doxycycline, levofloxacin, sulfamethoxazole/trimethoprim, and moxifloxacin are also ingredients of the ISS medical inventory [51] and can thus be used for treatment of bacterial infections aboard. In our tests (see Table 4), the environmental isolates from ISS were susceptible to amoxicillin (with clavulanic acid), ciprofloxacin, doxycycline, levofloxacin, sulfamethoxazole/trimethoprim, and moxifloxacin. We confirmed that ceftriaxone is not very effective against *Bacillus* sp., but it was effective against most non spore-forming isolates. When comparing the measured clindamycin MICs to resistance breakpoints defined for other species, 16 of 19 isolates could be rated potentially resistant against clindamycin: For all aerobic microorganisms, for which the clindamycin resistance breakpoint is defined in the EUCAST breakpoint table v6.0, it is “>0.5 μg/ml” (e.g., *Staphylococcus* sp. or *Corynebacterium* sp.). However, due to missing specific resistance breakpoints for non-pathogenic microbial isolates, this finding cannot be used for risk estimations.

The cultivable diversity of our older dust samples (17 bacterial species) was found to be lower than the cultivable diversity of the US American study (26 bacterial species), although a broader variety of cultivation media was used. In both studies, the genus *Bacillus* was the most prominent genus. However, on species level, no overlap between the isolates of these studies was found, which strongly indicates a difference in the microbial communities of the analyzed samples—either caused by longer storage or an overall difference in the microbiota composition of the Russian modules in 2004/2008 and of the US American modules in 2008–2012 (US American HEPA filter was installed from 2008 to 2011 and US American vacuum cleaner samples were taken 2012 [4]). Because of the use of nystatin in our study, we isolated only one eukaryotic isolate, *U. botrytis*, retrieved from medium with a pH of 9. *U. botrytis* was also not among the ten different fungi isolated by Checinska et al. 2015 (Additional file 3: Table S3).

Besides the analysis of the isolates, we carried out a comprehensive sequencing study to shed light onto the microbial diversity that was present 8–12 years ago in the Russian ISS segment.

Notably, PMA treatment did not result in positive amplification of 16S rRNA genes, although cultivation efforts confirmed the presence of viable cells. Either those were present only in very low numbers, so that the PMA treatment resulted in DNA below detection limit, and/or the microbes were present as hardy spores. The latter observation is in accordance with the sequencing results showing a high proportion of Bacilli (17.4%) and Clostridia (13.7%), whose spores require a harsh DNA extraction method [52].

In order to increase the amount of available DNA in the samples, and possibly also to trigger spore germination, we incubated the samples in warm LB medium before DNA extraction. Although the shift of the microbial community caused by the incubation was found to be substantial, we were able to retrieve enough DNA for a positive amplification of 16S rRNA genes in all samples. Without incubation, only three out of five samples gave a positive signal.

Besides spore-formers, our dataset revealed a high presence of signatures belonging to human-associated bacteria, including *Pseudomonas* [53], *Acinetobacter* [54], *Sphingomonas* [55], and *Corynebacterium* [56], throughout all samples. Most of these microbes have been detected on the human skin (such as *Corynebacterium* and *Staphylococcus*) and in the human gut (such as the strictly anaerobic *Faecalibacterium*). These findings confirm that the indoor airborne microbial community is derived directly from the astronauts’ presence as discussed in other studies [4, 57, 58].

We also found signatures of other strictly anaerobic genera, such as *Anaerococcus* and *Anaerostipes*, which can, however, tolerate oxygen when dormant. An intensive study of all taxa revealed a high proportion of extremotolerant microorganisms such as (i) spore-forming bacilli, with known resistance against radiation, pressure, desiccation, and space and Mars-simulation conditions [59–63] (and references therein), (ii) signatures of *Rhodococcus*, known for high resistance against desiccation and ultraviolet radiation [64], and (iii) members of *Cyanobacteria*, which are considered to be highly resistant against extreme conditions [65] (and references therein). The presence of extremotolerant microorganisms is also reflected in the predicted metabolic capacities. A various range of predicted genes encoding features which help organisms to withstand extreme conditions such as the two-component-system, several transporters, iron acquisition, and antibiotic resistance could be detected and were distributed equally throughout all samples.

In order to gain insight into the overall microbial community of the ISS, we compared the microbial community of the US segment [4] and the Russian segment (our study) by performing a joint data analysis, using NGS raw reads from both studies, processed by DADA2. We observed a high dissimilarity in the microbial composition between both segments, potentially caused by the different location, sampling time frame or methods used to gather the data. However, in the US segment, the microbial community was also dominated by human-associated microorganisms and the same core taxa on phylum level (Actinobacteria, Firmicutes, and Proteobacteria), although with a different relative abundance. It should be mentioned that a deeper comparison of both settings was not possible, due to the different primers used in the US segment study, as well as the short reads obtained (~130 bp is considered borderline for proper classification; paired reads could not be stitched [4]).

The most striking difference found during our comparison of already available data and that from our new study was the presence of archaeal signatures in the Russian samples. Overall, the presence of Archaea on the ISS has not been reported before, or previous attempts to detect them were negative [4, 5]. Archaea are generally known to be widely distributed in extreme environments, and are specifically well-adapted to biotopes with energy constraints. In our samples, we mainly found *Nitrososphaera* signatures, belonging to a group of chemolithoauthotrophic, ammonia-oxidizing archaea, distributed in soil and hot springs, but also abundant on human skin [16, 66]. Interestingly, in spite of known mismatches in the used primers [16], *Nitrososphaera* was also detected with universal primers, indicating a high abundance in the dust samples.

Signatures of human-associated *Methanobrevibacter* could also be observed. *Methanobrevibacter* species are described as anaerobic, human gut commensals [67]. Notably, the (rare) presence of Woesearchaeota signatures can also be reported. Their detection has been occasionally described in samples from soils and aquatic environments [68, 69]. Castelle et al. analyzed their genomic potential, revealing a small genome size and limited metabolic capacities, which suggests that these Archaea might have a symbiotic or parastic lifestyle [70]. Although the detection of archaeal 16S rRNA gene signatures cannot inform on the role and activity of these microbes yet, we can state that the International Space Station is/was indeed populated by all three domains of life.

## Conclusions

The ISS dust microbiome analyzed in this study contained living, hardy microorganisms and showed the presence of archaeal signatures. Numerous resistance capabilities towards environmental stresses were either predicted on a molecular level or shown by retrieved isolates. It should be stressed, that, although these findings raise many questions and require discussion, the International Space Station is and has always been a safe workplace [71] and no severe infections or disease outbreaks have been reported thus far. The specific resistance capacities of our non-pathogenic ISS isolates against desiccation, heat-shock, and some antibiotic compounds refer to samples that have been collected around a decade ago, and the findings need to be reconfirmed with novel microbial isolates. Nevertheless, it remains without doubt that microorganisms on the ISS experience selective pressures and that a number of microbes adapt to these stresses. Our findings and those of previous publications in this regard can now be considered for the planning of future, crewed long-term spaceflights, but also for potential habitats on the moon or other planetary bodies.

## Additional files

Additional file 1: Table S1. DADA2 output including negative controls (universal primer set); Highlighted RSVs were also found in the negative controls "RISS-LB-incubation-NC" and "RISS-protocol-NC" and were therefore removed from the dataset for further analysis. (XLSX 212 kb)
Additional file 2: Table S2. DADA2 output including negative control (archaeal primer set); After chimera filtering (see the “Bioinformatical analysis and data processing” section), no reads remained in the negative control (“RISSArch-protocol-NC”). (XLSX 8 kb)
Additional file 3: Table S3. Comparison of isolates from this study and Checinska et al. (2015). (DOCX 15 kb)
Additional file 4: Supplementary Figures. **Figure S1 A+B.** Resistance tests: minimal inhibitory concentration for 19 isolates as measured for 12 different antibiotics up to 256 μg/ml (A) or 32 μg/l (B). Horizontal lines show the non-species related breakpoints defined by EUCAST (Version 6.0, 2016). Above upper line: organism is resistant; below lower line: organism is sensitive; Y-axis shows the logarithmic concentration of the antibiotics as indicated on the Etest® reagent strips. **Figure S2.** Diversity of Archaea signatures in ISS samples. Displayed are observed taxa (richness), Shannon Index and InvSimpson Index. **Figure S3.** Differentially abundant genera in untreated and incubated samples. **Figure S4.** PcoA plot (unweighted Bray-Curtis distance) of US-ISS samples (US-ISS) and Russian ISS (RISS) samples. It has to be emphasized that only forward reads of both studies were processed here. Based on this approach, a clear, distinct clustering of Russian ISS samples and US-ISS samples is observed, which indicates dissimilarity in the microbial composition. (PPTX 477 kb)
Additional file 5: Table S4. DADA2 output (archaeal primer set; negative controls removed). (XLSX 8 kb)
Additional file 6: Table S5. DADA2 output (universal primer set; negative controls removed). (XLSX 192 kb)
Additional file 7: Table S6. Comparison of the ISS microbial community of archived dust samples (this study; “Dust filter and “Dust collector” samples) and freshly retrieved samples (Checinska et al. 2015; “HEPA-debris” and “vacuum-cleaner-bag-debris” samples). (XLSX 60 kb)
