# Supplementary material for: Resilient microorganisms in dust samples of the International Space Station—survival of the adaptation specialists
Source: Microbiome. 2016 Dec 20;4:65. doi: 10.1186/s40168-016-0217-7 (PMC5175303; doi:10.1186/s40168-016-0217-7)
Supplement: Additional file 3: Table S3. — Comparison of isolates from this study and Checinska et al. (2015). (DOCX 15 kb) [file 40168_2016_217_MOESM3_ESM.docx]

Supplementary table 3: Species isolated from the International Space Station in Checinska et al. 2015[4] and this study.

| Isolates of Checinska et al. 2015 [4] | Isolates of this study |
| --- | --- |
| **Prokaryotes - Bacteria** | **Prokaryotes - Bacteria** |
| **Actinobacteria** | **Actinobacteria** |
| *Agrococcus jenensis*  *Agrococcus lahaulensis*  *Arthrobacter agilis*  *Dietzia lutea*  *Kocuria rosea*  *Microbacterium foliorum* | *Brevibacterium halotolerans*  *Micrococcus yunnanensis* |
| **Alphaproteobacteria** | **Alphaproteobacteria** |
| *Brevundimonas vesicularis* | *Bradyrhizobium erythrophlei*  *Methylobacterium tardum* |
| **Betaproteobacteria** | **Betaproteobacteria** |
| *Masillia* sp. | *Cupriavidus metallidurans* |
| **Gammaproteobacteria** |  |
| *Pseudomonas luteola* |  |
| **Bacteroidetes** |  |
| *Pontibacter populi* |  |
| **Firmicutes** | **Firmicutes** |
| *Bacillus* anthracis-cereus group  *Bacillus aquimaris*  *Bacillus arbutinivorans*  *Bacillus firmus*  *Bacillus fusiformis*  *Bacillus ginsengihumi*  *Bacillus megaterium*  *Bacillus* sp.  *Brevibacillus* sp.  *Paenibacillus* sp.  *Paenibacillus taichungensis*  *Solibacillus silvestris*  *Staphylococcus cohnii*  *Staphylococcus epidermidis*  *Staphylococcus hominis*  *Staphylococcus warneri* | *Bacillus aerophilus*  *Bacillus clausii*  *Bacillus licheniformis*  *Bacillus pumilus*  *Bacillus safensis*  *Bacillus sonorensis*  *Bacillus* sp.  *Bacillus subtilis*  *Bacillus thermoamylovorans*  *Bacillus timonensis*  *Paenibacillus campinasensis*  *Salinibacillus aidingensis* |
| **Eukaryotes - Fungi** | **Eukaryotes - Fungi** |
| *Aspergillus niger*  *Aspergillus* sp.  *Aspergillus terreus*  *Aspergillus unguis*  *Glomus* sp.  *Mucor* sp.  *Penicillium camemberti*  *Penicillium* sp.  *Rhodotorula* sp.  Unidentified fungi | *Ulocladium botrytis* |
