# Supplementary material for: Resilient microorganisms in dust samples of the International Space Station—survival of the adaptation specialists
Source: Microbiome. 2016 Dec 20;4:65. doi: 10.1186/s40168-016-0217-7 (PMC5175303; doi:10.1186/s40168-016-0217-7)
Supplement: Additional file 4: — Supplementary Figures. Figure S1 A+B. Resistance tests: minimal inhibitory concentration for 19 isolates as measured for 12 different antibiotics up to 256 μg/ml (A) or 32 μg/l (B). Horizontal lines show the non-species related breakpoints defined by EUCAST (Version 6.0, 2016). Above upper line: organism is resistant; below lower line: organism is sensitive; Y-axis shows the logarithmic concentration of the antibiotics as indicated on the Etest® reagent strips. Figure S2. Diversity of Archaea signatures in ISS samples. Displayed are observed taxa (richness), Shannon Index and InvSimpson Index. Figure S3. Differentially abundant genera in untreated and incubated samples. Figure S4. PcoA plot (unweighted Bray-Curtis distance) of US-ISS samples (US-ISS) and Russian ISS (RISS) samples. It has to be emphasized that only forward reads of both studies were processed here. Based on this approach, a clear, distinct clustering of Russian ISS samples and US-ISS samples is observed, which indicates dissimilarity in the microbial composition. (PPTX 477 kb) [file 40168_2016_217_MOESM4_ESM.pptx]

## Slide 1
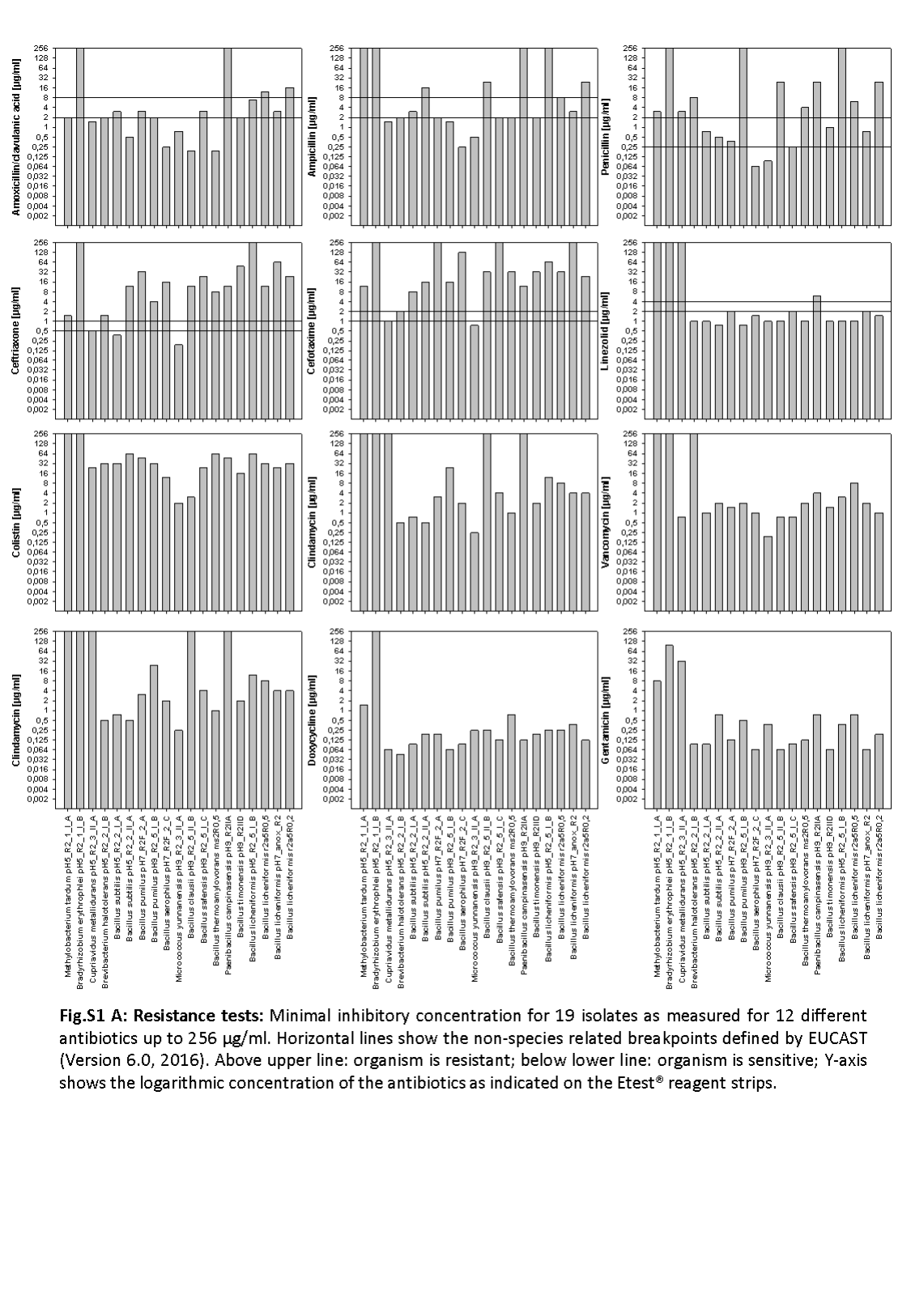

## Slide 2
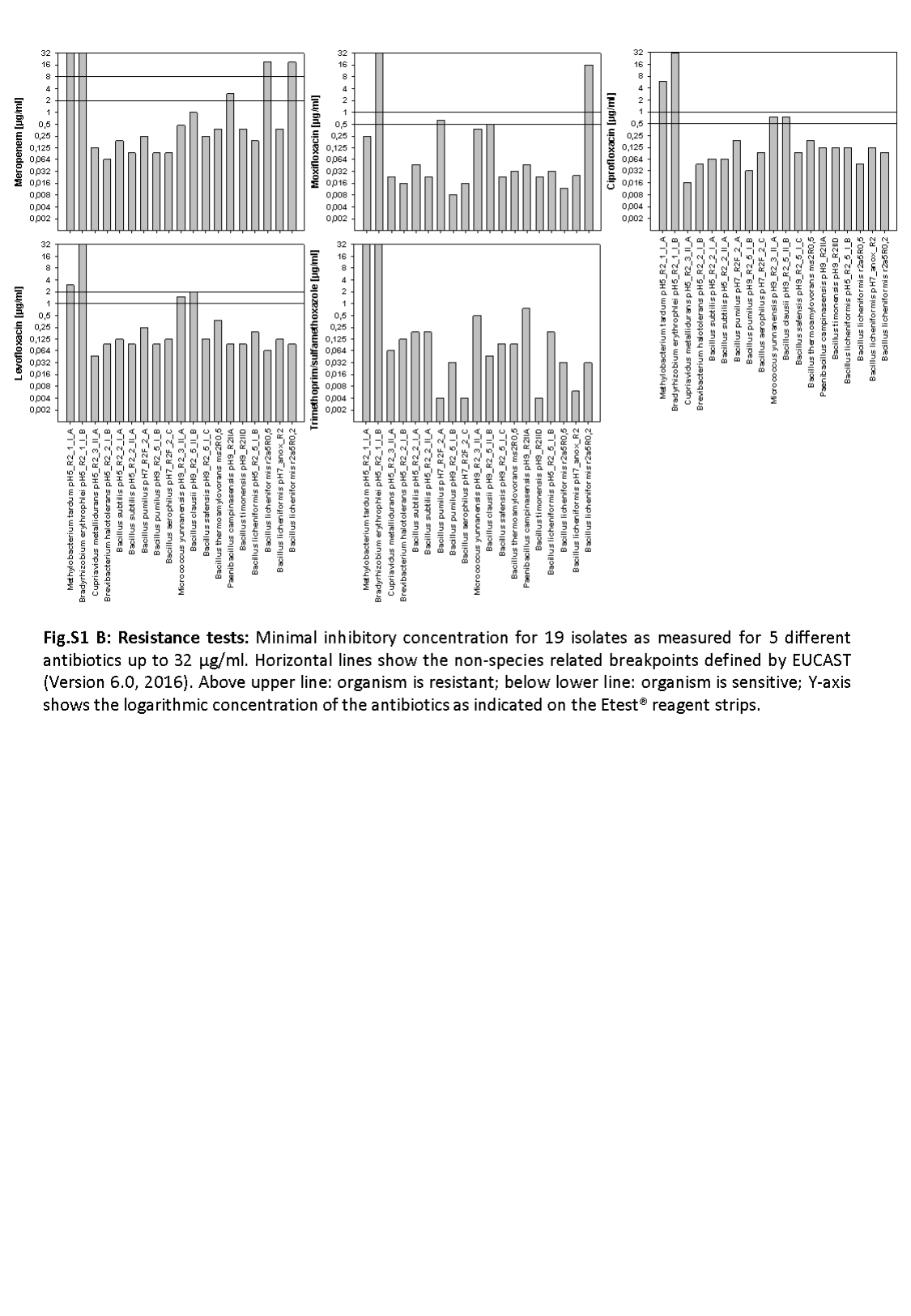

## Slide 3
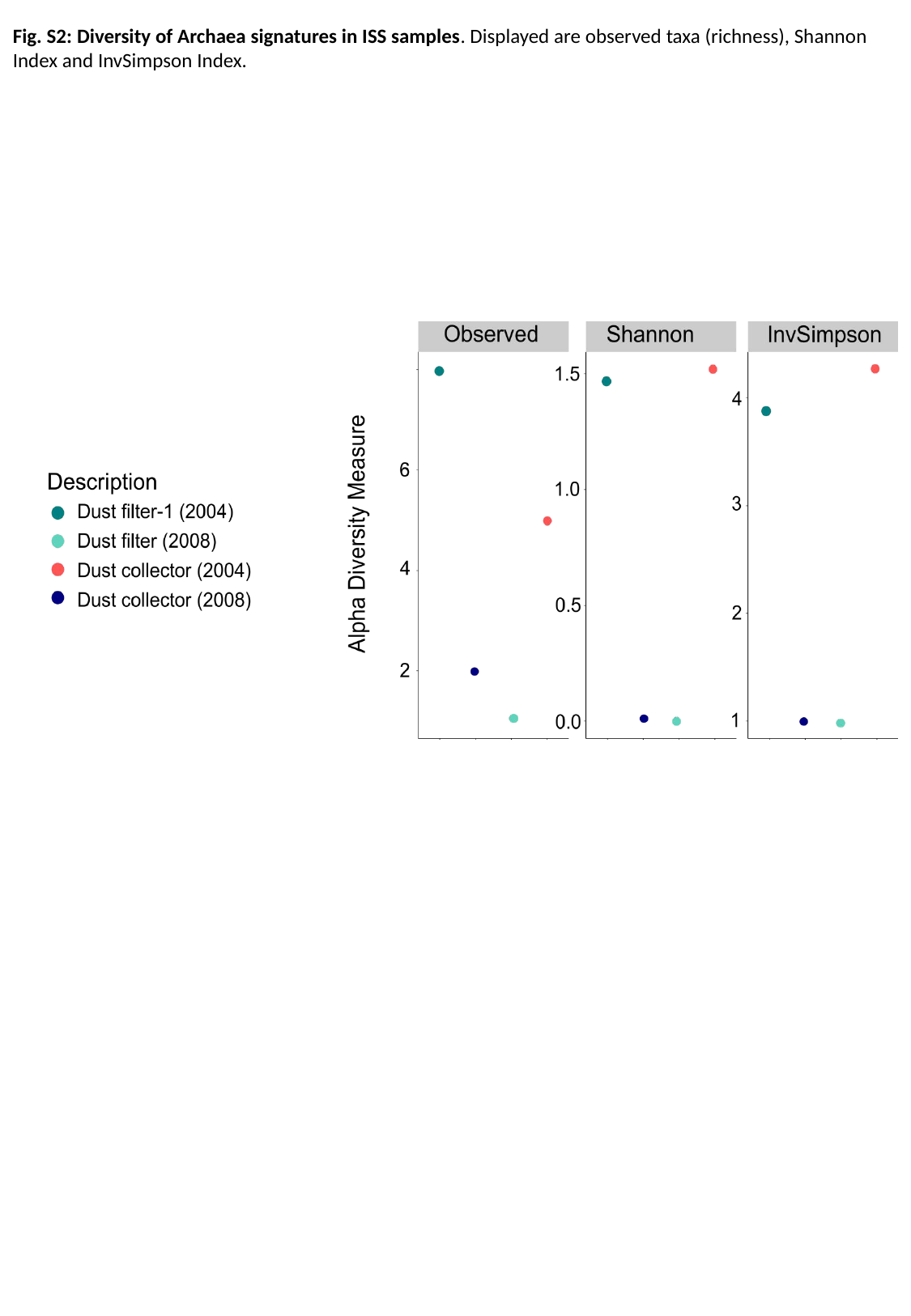

Fig. S2: Diversity of Archaea signatures in ISS samples. Displayed are observed taxa (richness), Shannon Index and InvSimpson Index.

## Slide 4
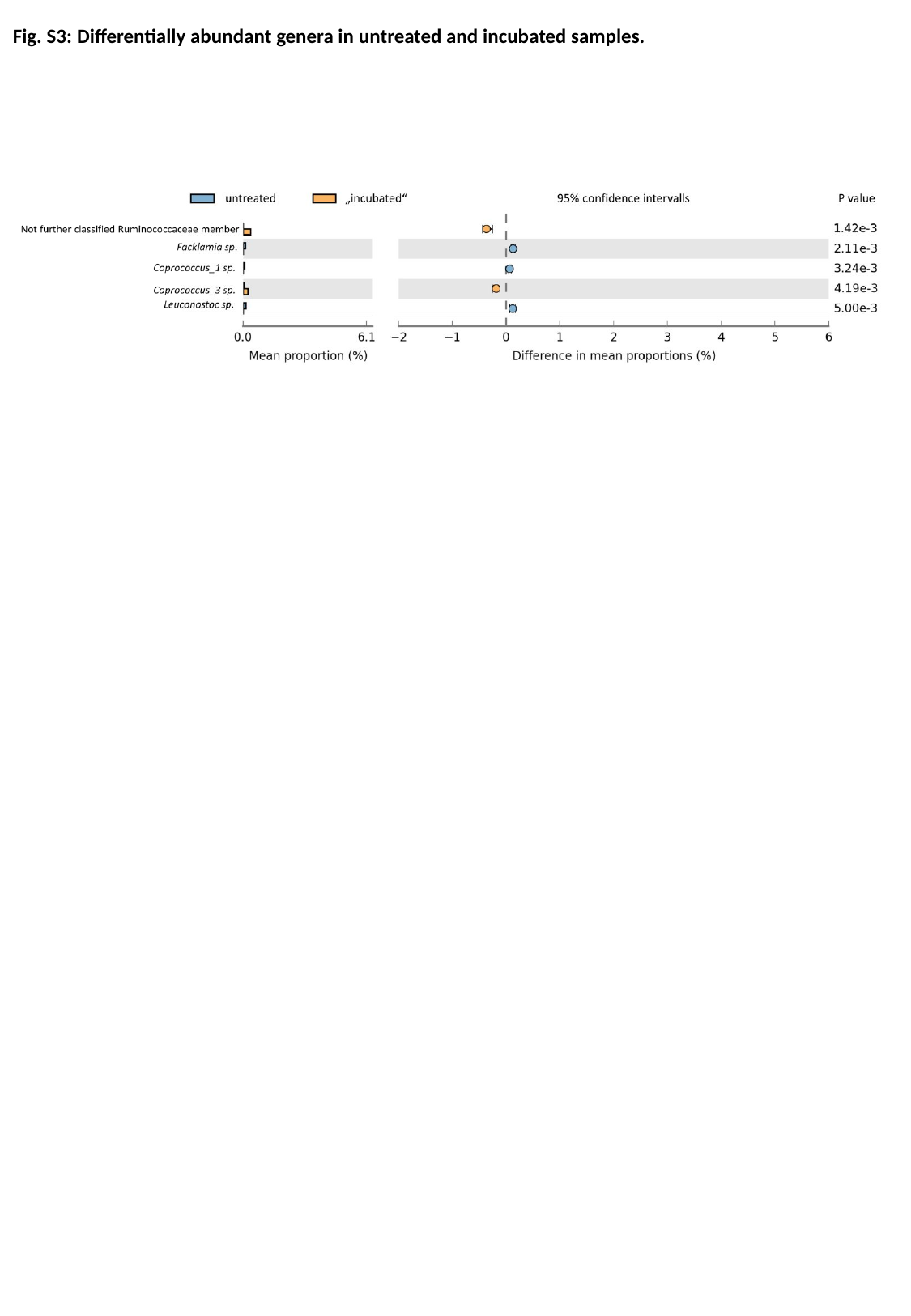

Fig. S3: Differentially abundant genera in untreated and incubated samples.

## Slide 5
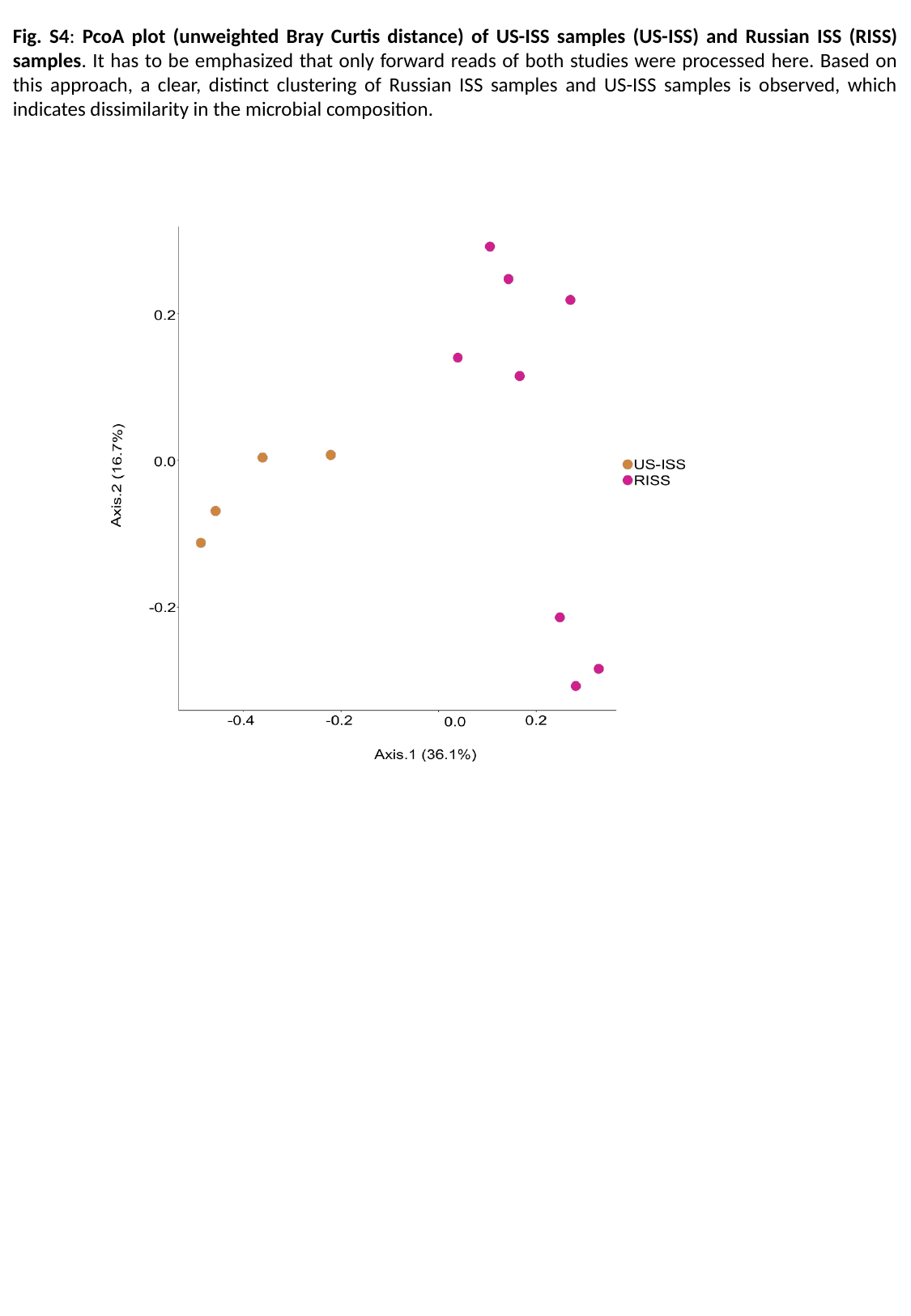

Fig. S4: PcoA plot (unweighted Bray Curtis distance) of US-ISS samples (US-ISS) and Russian ISS (RISS) samples. It has to be emphasized that only forward reads of both studies were processed here. Based on this approach, a clear, distinct clustering of Russian ISS samples and US-ISS samples is observed, which indicates dissimilarity in the microbial composition.
